# Profiling of Oral Microbiota in Early Childhood Caries Using Single-Molecule Real-Time Sequencing

**DOI:** 10.3389/fmicb.2017.02244

**Published:** 2017-11-15

**Authors:** Yuan Wang, Jie Zhang, Xi Chen, Wen Jiang, Sa Wang, Lei Xu, Yan Tu, Pei Zheng, Ying Wang, Xiaolong Lin, Hui Chen

**Affiliations:** Department of Conservative Dentistry and Periodontics, Affiliated Hospital of Stomatology, College of Medicine, Zhejiang University, Hangzhou, China

**Keywords:** caries, oral microbiome, PacBio RS II system, sequencing, healthy, NCT02341352. Registered 13 January 2015. retrospectively registered.

## Abstract

**Background:** Alterations of oral microbiota are the main cause of the progression of caries. The goal of this study was to characterize the oral microbiota in childhood caries based on single-molecule real-time sequencing.

**Methods:** A total of 21 preschoolers, aged 3–5 years old with severe early childhood caries, and 20 age-matched, caries-free children as controls were recruited. Saliva samples were collected, followed by DNA extraction, Pacbio sequencing, and phylogenetic analyses of the oral microbial communities.

**Results:** Eight hundred and seventy six species derived from 13 known bacterial phyla and 110 genera were detected from 41 children using Pacbio sequencing. At the species level, 38 species, including *Veillonella* spp., *Streptococcus* spp., *Prevotella* spp., and *Lactobacillus* spp., showed higher abundance in the caries group compared to the caries-free group (*p* < 0.05). The core microbiota at the genus and species levels was more stable in the caries-free micro-ecological niche. At follow-up, oral examinations 6 months after sample collection, development of new dental caries was observed in 5 children (the transitional group) among the 21 caries free children. Compared with the caries-free children, in the transitional and caries groups, 6 species, which were more abundant in the caries-free group, exhibited a relatively low abundance in both the caries group and the transitional group (*p* < 0.05). We conclude that *Abiotrophia* spp., *Neisseria* spp., and *Veillonella* spp., might be associated with healthy oral microbial ecosystem. *Prevotella* spp., *Lactobacillus* spp., *Dialister* spp., and *Filifactor* spp. may be related to the pathogenesis and progression of dental caries.

## Introduction

Caries is one of the most common infectious diseases that occurs in children who consume refined carbohydrate-rich diets (Bradshaw and Lynch, 2013). Caries is a destructive process leading to sustained decalcification of tooth enamel and dentin (Andlaw, 1960). When the infection has progressed enough to allow bacteria to overwhelm the dental pulp tissue, a toothache can result, and if left untreated, the pain worsens with exposure to heat, cold, or sweet foods and drinks (Marcenes et al., 2013). In highly progressed cases, infection can spread from the affected tooth to the surrounding soft tissues, resulting in complications such as phylogenetic osteomyelitis (Baur et al., 2012) and bacterial endocarditis (Gundre et al., 2011), which can be life threatening. Once caries occurs, the damage to teeth is irreversible; thus, preventive intervention of caries in children is of particular clinical significance (2009). A systematic analysis of the Global Burden of Disease Study in 2010 showed that the global prevalence of dental caries of permanent teeth was up to 35.29% (Vos et al., 2012). In addition to diet and host factors, the occurrence and development of dental caries is closely related to the imbalance of the oral microbiota (Yang et al., 2012). Acidogenic and acidophilic microorganisms play an important role in the etiology of the disease (Yang et al., 2012).

In common with the microbiota in other parts of the human body, the role of the oral microbiota has been a research hotspot for a very long history (Crawford and Shankle, 1961). The Human Microbiome Project (HMP), which was launched by the NIH in 2007, provided the first glimpse of the microbial diversity of healthy humans, both in the oral cavity and other anatomical sites (Human Microbiome Project, 2012a,b). Numerous comparative studies have shown that oral microbiotas exhibit significant differences in the relative abundance of various genera, families, or phyla in childhood caries (Yang et al., 2012; Teng et al., 2015). In 2010, Ling et al. compared the difference in oral bacterial diversity between caries and caries-free children using next-generation sequencing (NGS) technology (Ling et al., 2010). Subsequent studies investigated the shifting bacterial profiles in different caries states (Jiang et al., 2013, 2014). However, the short reads generated by the second-generation sequencing platform introduce biases depending on which variable regions are applied and cannot provide effective resolution below the bacterial genus level, limiting the oral microbial ecology studies in caries.

Recent studies have shown that one of the most significant issues with NGS is the difficulty of assembling short-length reads (Orkunoglu-Suer et al., 2015). Thus, in spite of the low cost and extremely high throughput, the NGS platform, including Roche 454 and MiSeq, is sometimes less accurate as a result of short read lengths (Mosher et al., 2014). Operational taxonomic units (OTUs) has been an operational definition used to classify groups of closely related bacteria, however, the short-read approach by NGS introduces biases depending on which variable regions are used and cannot provide effective resolution below the bacterial genus level. The PacBio RS II platform, a newly emerging third-generation DNA sequencer produced by Pacific Biosciences, Inc., is based on single-molecule real-time (SMRT) DNA sequencing system that provides the highest consensus accuracy and longest read lengths of any available sequencing technology (Eid et al., 2009; Otto, 2011; Jiao et al., 2013; Zhang et al., 2015). Although, the SMRT has been applied to the lung microbiota (Toma et al., 2014) and some environmental samples (Mosher et al., 2014), its use in oral microbiota research is still an open area.

In 2013, Mosher and colleagues reported that the PacBio RS SMRT sequencing platform with XL/C2 chemistry revealed higher error rates when compared with the Roche 454 GS FLX chemistry (Mosher et al., 2013). One year later the same group reanalyzed the previously constructed libraries with the updated PacBio RS II system with P4/C2 chemistry. The results show that the PacBio sequencing platform surpassed the accuracy of Roche/454 pyrosequencing platform (Mosher et al., 2014). A recent study show that the PacBio sequencing error rate is now in the same range of the previously widely used Roche 454 sequencing platform and current MiSeq platform, since errors can be effectively reduced by consensus while building contigs from the reads (Wagner et al., 2016).

Our unpublished data have shown that PacBio outperformed other sequencers, such as Roche 454 and MiSeq, in terms of the length of reads, and it reconstructed the greatest portion of the 16S rRNA genome when sequencing the oral microbiota. At the genus level, 0.68% of genera were unknown or unclassified using the MiSeq platform, whereas on the PacBio platform, all of the microorganisms were classified. At the species level, 50.3% of oral bacteria were unknown or could not be classified by the MiSeq platform, but only 0.03% of microorganisms were unidentified when using the PacBio RS II platform. Therefore, compared to NGS, the PacBio platform can establish a higher estimate of richness and identify organisms at a higher resolution (Mosher et al., 2014). Thus, SMRT sequencing is more appropriate for studies of the relationship between the microbiota and related diseases on the species level.

From this point of view, further study of the oral microbiota by means of applying the SMRT technique is necessary for a deeper understanding the etiology of caries. Saliva is thought to be a mirror that reflects the oral microbial characteristics and various disease states in an individual (Lima et al., 2010). Therefore, salivary analysis has become an important resource for the evaluation of the oral microbiota with physiological and pathological implications, and it is a useful tool for disease diagnosis. Additionally, saliva collection is simple and non-invasive, and saliva is easy to store.

The aim of this study was to characterize the oral microbiota by comparing and analysing saliva from 20 children with caries and 21 caries-free children of Han Chinese origin based on the SMRT DNA sequencing system.

## Materials and methods

### Subjects

Forty-one children aged 54–74 months were analyzed and followed up for 6 months, including 21 who were free of caries (dmfs = 0) and 20 caries individuals (dmfs ≥ 11). The children were unrelated individuals of both genders, selected from the same kindergarten in the Hangzhou urban area. Their family economic status was below the average level of this region. Any subject who met the following criteria was excluded from the study: having fewer than 18 teeth, having received antibiotics or fluoride treatment in the previous 3 months, or having active bacterial or viral infections in other parts of the body.

Dental caries was diagnosed according to the criteria of the International Caries Detection and Assessment System II (ICDAS-II; Ismail et al., 2007) by comprehensive examinations by two professional dentists who were previously trained and calibrated for the evaluation and sampling procedures. According to the caries categories, ICDAS™ codes 0–2 were defined as caries-free, and ICDAS™ codes 3–6 were defined as caries. The dmfs index measures the number of decayed, missing and filled tooth surfaces in epidemiologic surveys of dental caries. It was adopted in this study to evaluate each child's caries status and thus to define and distinguish the caries-free children and the caries children. Researchers in this study have been trained for the standard biosecurity and institutional safety procedures.

### Ethical approval and informed consent

This study was carried out in accordance with the ethical committee of the Affiliated Hospital of Stomatology, School of Medicine, Zhejiang University (Approval Number: 2013-8). Written informed consent was obtained from the parents or guardians of all participants prior to enrolment, which is in accordance with the principles of the Declaration of Helsinki.

### Saliva sampling and isolation of bacterial DNA

The subjects were instructed neither to eat and drink nor to perform any oral hygiene procedure 2 h before sampling. Saliva samples were collected from all subjects in the morning between 9:00 and 11:00 a.m. Unstimulated saliva samples were collected according to a protocol, modified from a previous study (Ebersole et al., 2013). The children were initially asked to rinse their mouth mouths thoroughly with deionized water prior to sampling, followed by collection of at least 5 ml unstimulated saliva in a plastic cup. Finally, the samples were transferred into sterile cryogenic vials. Then the samples were placed into liquid nitrogen and stored at −80°C until use.

Bacterial DNA was extracted using the QIAamp DNA Mini Kit (Qiagen, Hilden, Germany) as previously described (Jiang et al., 2013, 2014). The enriched microbial DNAs were purified by ethanol precipitation. The DNA concentration was estimated by the absorbance ratios at A260/A280 and A260/A230 using a NanoDrop spectrophotometer (Thermo Elactron corporation, USA), and molecular size was estimated by agarose gel electrophoresis. The DNAs were stored at −20°C until use.

### PCR amplification of the 16S rRNA gene

PCR amplification of the 16S rRNA gene hypervariable V1–V9 regions was performed with bacterial primers 27F (5′-AGAGTTTGATCCTGGCTCAG-3′) and 1492R (5′-GGTTACCTTGTTACGACTT-3′). The products were extracted with the AxyPrep DNA Gel Extraction kit (Axygen, USA) and were then examined by agarose gel electrophoresis. Based on the electrophoretic results, the PCR products were quantified by Quantifluo™-ST (Promega, USA). The products from different samples were then mixed at equal ratios for pyrosequencing between the two groups.

### DNA library construction and sequencing

**Figure 1:**
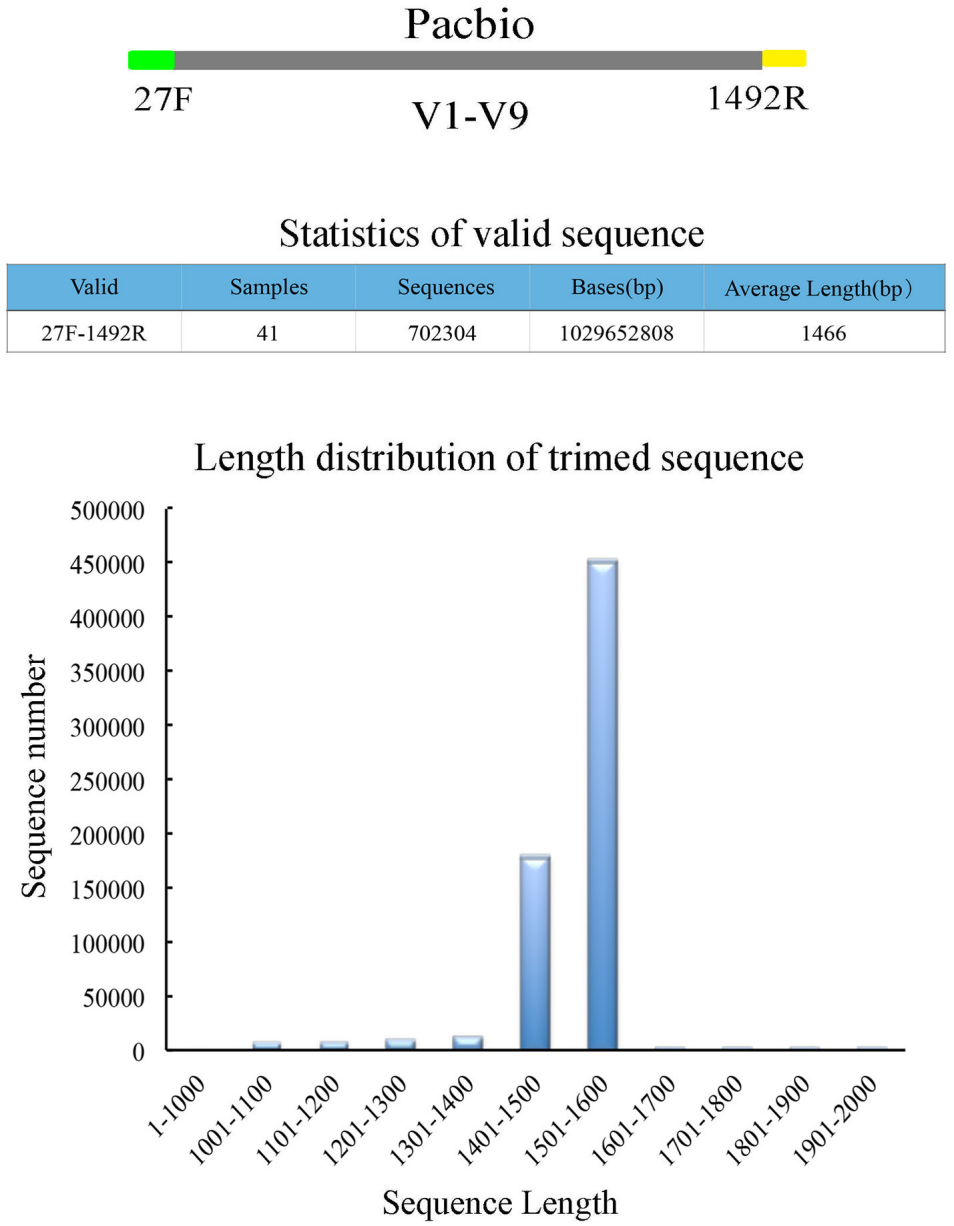
Sequencing results of full-length 16S rRNA amplicons (V1–V9) from 41 oral samples.

Purified amplicons were sequenced using Single Molecule Real Time (SMRT) technology on a PacBio RS II sequencer. DNA library construction was performed following the manufacturer's instructions (PacBio). Barcoded 16S rRNA amplicons (V1–V9 hypervariable region) of all samples were sequenced using the PacBio RS II platform. A total of 11 samples were placed per SMRT cell for amplicon sequencing. In order to decrease the sequencing error rate, Pacbio circular consensus sequencing (CCS) reads were derived from the multiple alignments of sub-reads. In CCS, the DNA polymerase reads a ligated circular DNA template multiple times, which can effectively generate a consensus sequence from multiple reads of a single molecule (Travers et al., 2010; Wagner et al., 2016). Raw sequences were initially processed through the PacBio SMRT portal. Sequences were filtered for a minimum of 3 passes, and a minimum predicted accuracy of 99% (minfullpass = 3, minPredictedAccuacy = 99). The predicted accuracy of 99%, which is defined as the threshold below which a CCS is considered as noise. The files generated by the PacBio platform were then used for amplicon size trimming to remove sequences outside the expected amplicon size (<1,000 and >2,000 bp). Sequencing adapters and low-quality sequences were then filtered out using the software package of Mothur version (v.1.30.1; Schloss et al., 2009). UCHIME is used for detection and removal of chimeric sequences with two or more segments (Edgar et al., 2011). Finally, the high quality filtered sequences were generated for the downstream analysis (Figure 1). Sequence files and metadata for all samples used in this study have been deposited in SRA (PRJNA388240; SRP108162).

### OTU profile analysis

The high-quality reads were aligned in accordance with Silva alignment protocols and clustered into OTUs (Pruesse et al., 2007; Quast et al., 2013).

QIIME (Caporaso et al., 2010) was used for OTU clustering, and OTUs that reached the 97% similarity level were used for analyses of alpha-diversity (Shannon index and Simpson index), richness (Ace index and Chao index), and coverage, as well as rarefaction curves, by using Mothur software (Schloss et al., 2009; version v.1.30.1).

The Chao index was calculated as follows:

(1) $$\begin{array}{r} S_{\text{chao}1}=S_{0bs}+\frac{n_{1}\left(n_{1}-1\right)}{2\left(n_{2}+1\right)} \end{array}$$

*S*_chao1_ is the number of OTU estimated
*S*_obs_ is the number of OTU actually generated
*n*_1_ is the OTU with only 1 reads (e.g., “singletons”)
*n*_2_ is the OTU with only 2 reads (e.g., “doubletons”)

The Ace index was calculated as follows:

$$\begin{array}{l} S_{ACE}=\{\begin{array}{l} S_{abund}+\frac{S_{rare}}{C_{ACE}}+\frac{n_{1}}{C_{ACE}}\gamma_{ACE}^{\wedge^{2}},\;for\;\gamma_{ACE}^{\wedge^{2}}<0.80\\ S_{abund}+\frac{S_{rare}}{C_{ACE}}+\frac{n_{1}}{C_{ACE}}\gamma_{ACE}^{\sim 2},\;for\;\gamma_{ACE}^{\wedge^{2}}\geq 0.80 \end{array}\\ C_{ACE}=1-\frac{n_{1}}{N_{rare}},\;\;N_{rare}=\sum_{i=1}^{abund}in_{i}\\ \gamma_{ACE}^{\wedge^{2}}=max\left[\frac{S_{rare}\sum_{i=1}^{abund}i(i-1)n_{i}}{C_{ACE}\;N_{rare}(\;N_{rare}-1)}-1,0\right]\\ \gamma_{ACE}^{\sim 2}=\\ max\left[\gamma_{ACE}^{\wedge^{2}}\left\{1+\frac{N_{rare}(1-C_{ACE})\sum_{i=1}^{abund}i(i-1)n_{i}}{\;N_{rare}(\;N_{rare}-C_{ACE})}-1\right\},0\right] \end{array}$$

*n*_i_ is the number of OTU which contains *n* reads
*S*_*rare*_ is the number of OTU which contains “abund” reads or less
*S*_*abund*_ is the number of OTU which contains more than “abund”
abund is the threshold value of OTU, which defaults to 10.

The species-level OTUs and relative richness of phyla, classes, orders, families, genera and species for each sample between the caries and caries-free groups were compared. Phylotypes with median relative abundances >0.01% in either the caries or caries-free groups were included for comparison.

### Statistical analysis

One-way analysis of variance (ANOVA) test was used to compare difference in diversity indices and multiple comparison between the groups was performed using Student–Newman–Keuls (S-N-K) test. Wilcoxon signed rank test was used for bacterial relative abundance at different levels. *P* < 0.05 was considered statistically significant difference among species. Graphical outputs were performed using customized R scripts and SPSS for Windows (version 19.0; SPSS Inc., Chicago, IL, USA).

## Results

### Sample collection, sequencing, and quality control

A total of 41 preschool children were enrolled in this study (Table S1). All subjects (including 20 who were caries and 21 caries-free) were divided into a caries group and a caries-free group according to dental examination results and follow-up information after 6 months (Table S2). Saliva samples were collected, and bacterial diversity was analyzed by employing single molecule real-time sequencing of 16S rRNA gene amplicons. After filtering out low quality reads and host contamination, a total of 702,304 post-trimming 16S rRNA gene reads were obtained, with 17,129 reads per sample on average (Table S3). The average read length was 1,466 bp, and 94.2% of all valid sequences were distributed between 1,400 and 1,600 bp (Figure 1).

### Diversity of saliva microbiota associated with caries

The 16S rRNA gene reads were then assigned to individual species-level OTUs at 3% dissimilarity. In total, 3,287 OTUs were detected in these 41 children, with 876 species derived from 13 known bacterial phyla and 110 genera. A total of 2,443 OTUs were found in the caries group, and 2,432 OTUs were found in the caries-free group. The number of OTUs in each sample between the caries and caries-free groups is shown in Figure S1. In total, 1,588 OTUs were common to both cohorts, representing 65.3 and 65.0% of the caries and caries-free groups, respectively. Eighty-three OTUs were found to be associated with caries, and 38 OTUs were enriched in the caries-free group (*p* < 0.05). This clearly indicated that considerable microfloral differences were present between the two cohorts.

The alpha-diversity indices were calculated from the OTU abundance profiles. The community diversity, Good's coverage and richness of the oral microbiota are shown in Table S4. From the comparisons of the community diversity indices (Shannon and Simpson index), it was found that the caries group displayed slightly higher diversity than the control group (Figure S2). However, there was no significant difference between the two groups (*p* > 0.05). Good's coverage was 100% for sequences in each of the samples, indicating that the 16S rRNA gene sequences identified in the two groups represented almost all the bacterial sequences present in the saliva samples.

**Figure 2:**
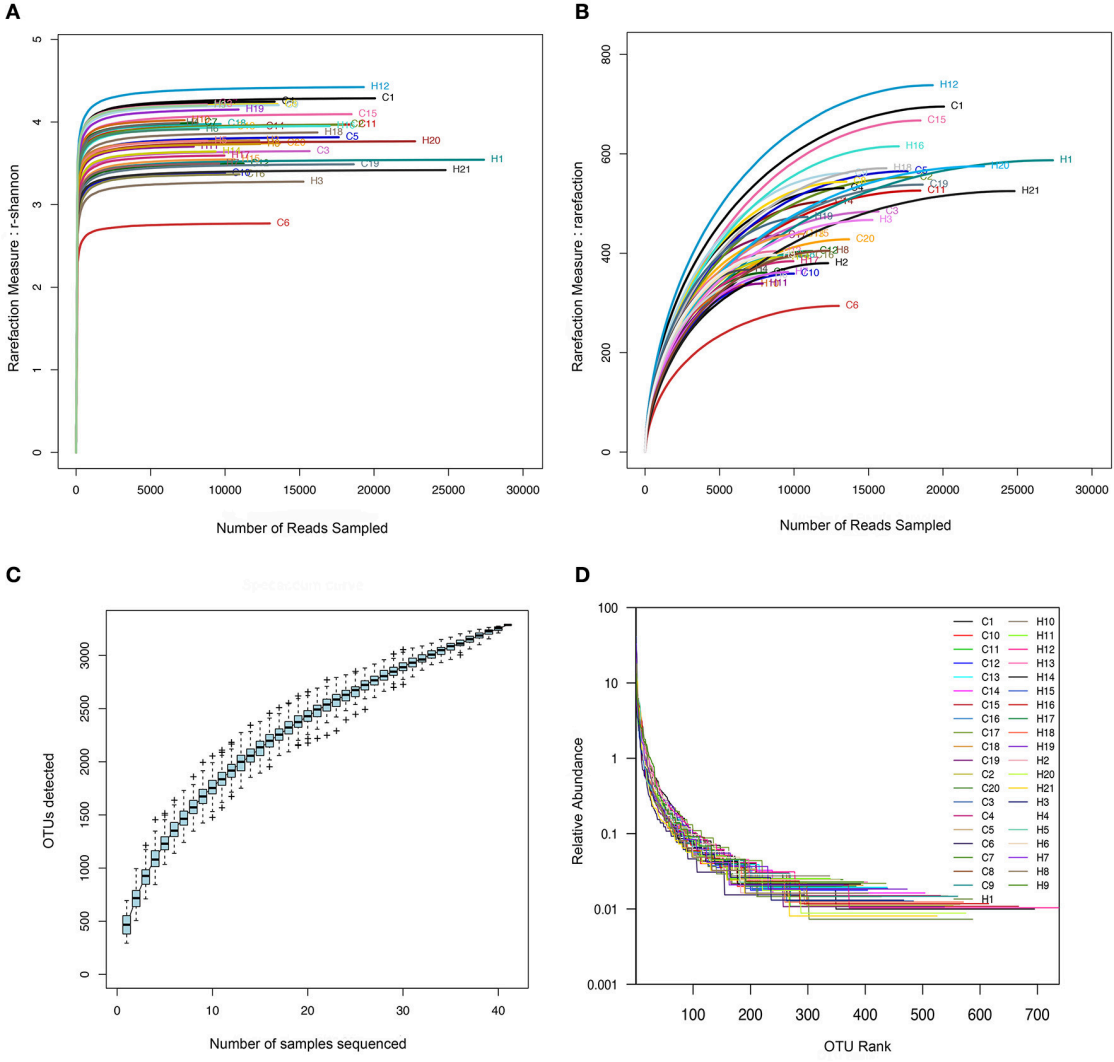
The rarefaction and extrapolation sampling curves based on the data from the 41 oral samples. (H for caries free group, C for caries group). **(A)** Shannon Wiener curves. **(B)** Rarefaction curves. **(C)** Specaccum curve. **(D)** Rank-abundance distribution curve.

The species richness of the oral microbiota in the saliva of each individual was estimated by richness indices (OTU number, Chao, and ACE index; Figure S3) and rarefaction analysis (Figure 2). A slightly higher richness was found in the children with caries, but there was no statistical significance (*p* > 0.05).

The shape of the rarefaction curves evidenced that a plateau was completely reached in both of the groups, and the same result was observed for the Shannon Wiener curves, indicating that microbial richness of the sampled saliva was almost completely sequenced at the current sequencing depth (Figures 2A,B). Comparisons of the rarefaction curves in the children with caries and the caries-free children revealed that the two groups displayed similar richness. We constructed Specaccum curves for each sample and for all OTUs detected to assess the current state of sampling. The saturated Specaccum curve indicated that the sampling (41 samples) was comprehensive (Figure 2C). A rank-abundance distribution curve for the oral microbial communities was constructed to visualize species richness and species evenness. According to this curve, the difference in the species evenness and richness was not significant (Figure 2D).

### Structure of caries-associated microbiota

**Figure 3:**
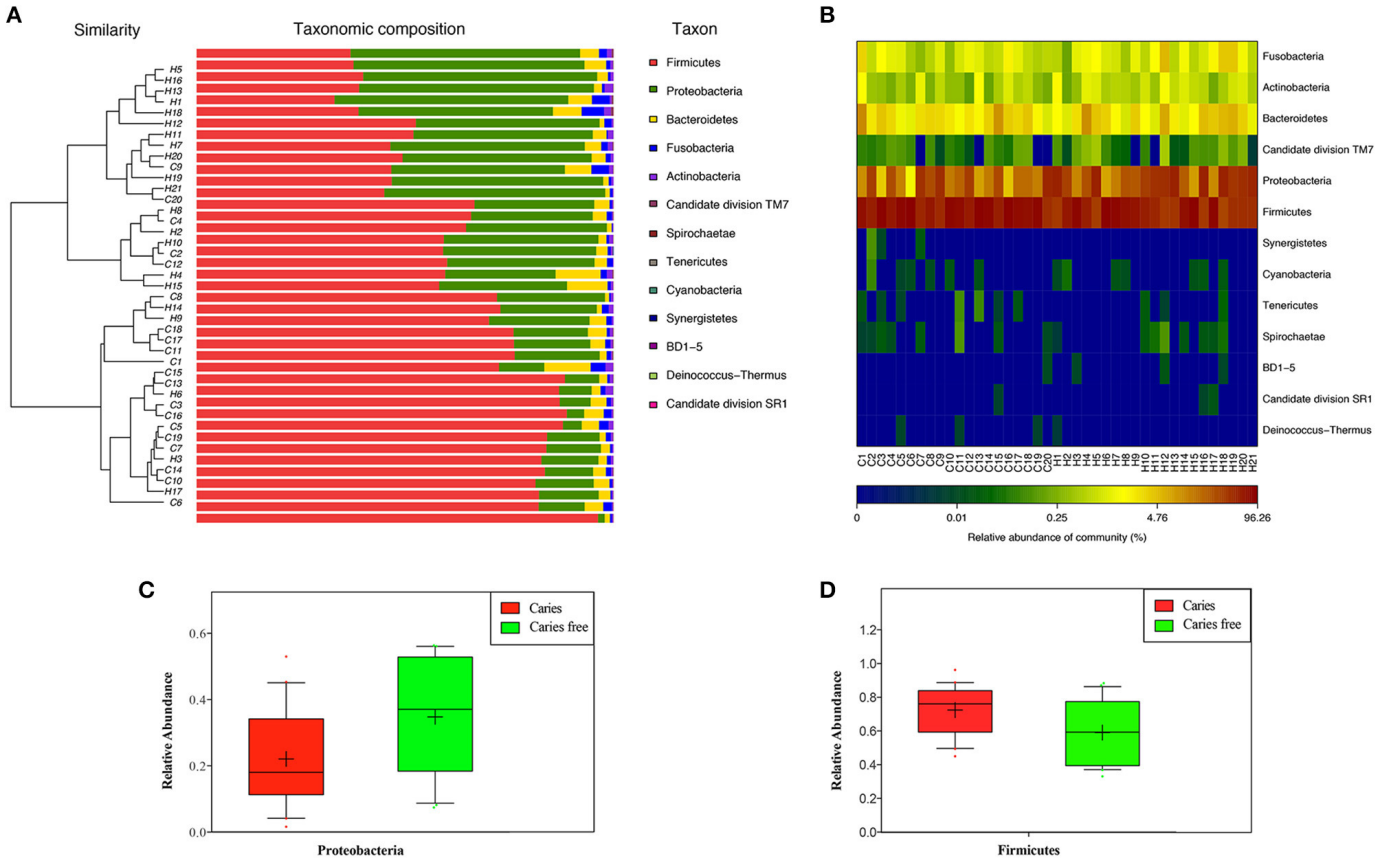
**(A)** Microbial community barplot with cluster tree at phylum level. **(B)** Relative abundance of phylotypes in each group at the phylum level. **(C)** Phylum that enriched in caries free group. **(D)** Phylum that enriched in caries group.

**Figure 4:**
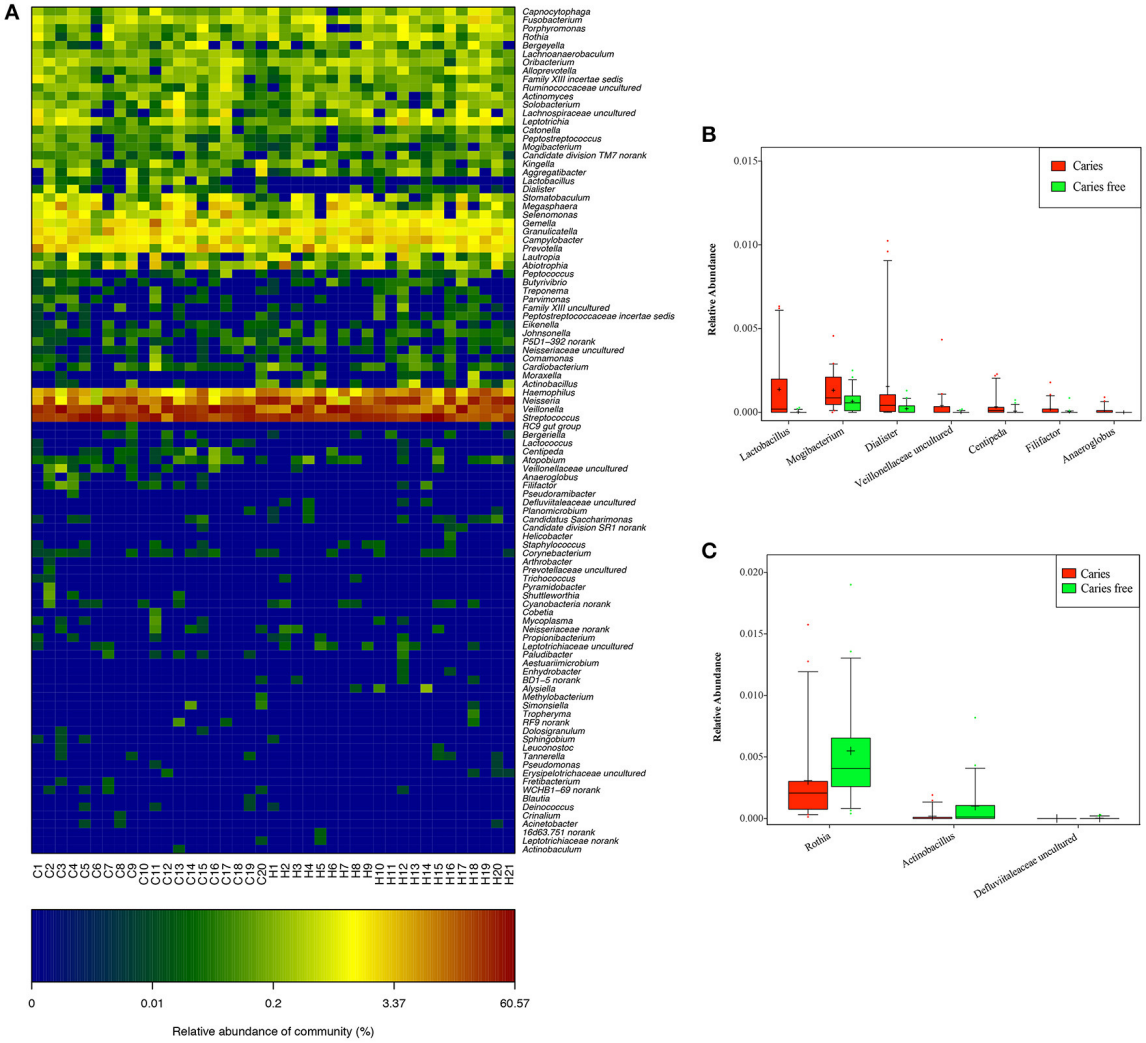
**(A)** Relative abundance of phylotypes in each group at the genus level. Genera that enriched in caries **(B)**, and caries free **(C)** groups.

**Figure 5:**
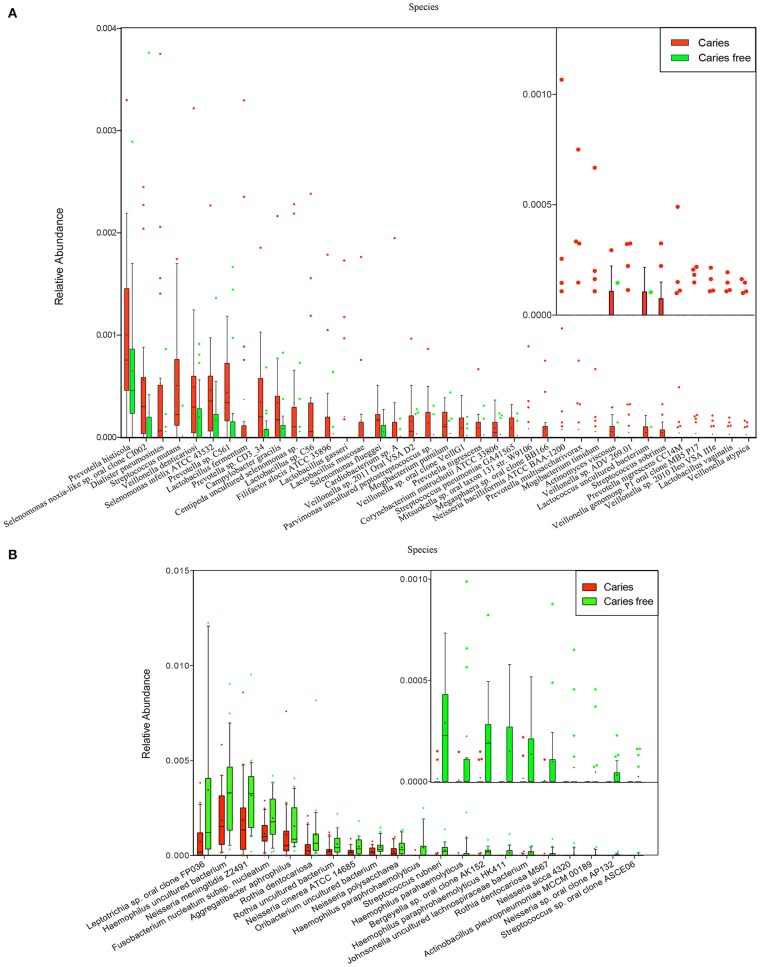
Species that enriched in caries **(A)**, and caries free **(B)** groups.

By analysing the saliva microbiota of children with and without caries, a total of 13 phyla, 110 genera, and 877 species were detected. The overall structure of the oral microbiota for each group is shown in Figures 3–**5**. The relative abundance of phyla, classes, orders, families, genera, and species between the caries and caries-free cohorts were compared, and any phonotype with a median relative abundance below 0.01% was excluded.

The most abundant phylum was Firmicutes, followed by Proteobacteria, Bacteroidetes, Fusobacteria, Actinobacteria, and TM7, which represented 99.97 and 99.98% of the oral microbiota in children with and without dental caries, respectively (Figure S4). Compared with the caries-free controls, children with caries had fewer Proteobacteria but higher levels of Firmicutes (fdr < 0.05; Figure 3).

At the genus level, *Streptococcus, Veillonella, Neisseria, Haemophilus*, and *Prevotella* dominated the communities in both groups (Figure S4). A total of 7 bacterial genera, including *Lactobacillus, Mogibacterium, Dialister, Veillonellaceae uncultured, Centipeda, Filifactor*, and *Anaeroglobus*, showed higher abundance in the caries group, whereas 3 genera, including *Rothia, Actinobacillus, and Defluviitaleaceae uncultured*, had lower abundance (Figure 4).

At the species level, *Veillonella uncultured bacterium, Streptococcus mitis, Streptococcus salivarius* subsp. *thermophilus, Neisseria flavescens*, and *Neisseria uncultured bacterium* were found at relatively high abundances in both the children with caries and the caries-free children (Figure S4). The subjects with caries exhibited relatively increased abundance of 38 species, including *Veillonella* spp. (*Veillonella atypical* and *Veillonella denticariosi*, etc.), *Streptococcus* spp. (*Streptococcus mutans* and *Streptococcus sobrinus*, etc.), *Prevotella* spp. (*Prevotella histicola, Prevotella multisaccharivorax*, and *Prevotella nigrescens*, etc.), *Lactobacillus* spp. (*Lactobacillus fermentum, Lactobacillus gasseri*, and *Lactobacillus mucosae*, etc.), *Selenomonas* spp., *Mogibacterium* spp., *Actinomyces viscosus*, and *Dialister pneumosintes*, etc. (Figure 5).

Compared with the children with caries, in the caries-free children, there were 21 abundant species, including *Neisseria* spp. (*Neisseria cinerea ATCC 14685* and *Neisseria polysaccharea*, etc,), *Haemophilus* spp. (*Haemophilus parahaemolyticus* and *Haemophilus paraphrohaemolyticus*, etc,), *Streptococcus* spp., *Rothia* spp., and *Aggregatibacter aphrophilus*, suggesting that the oral microbial community differs in phylogenetic structure between children with caries and caries-free children (Figure 5).

Across a range of species that exhibited statistically significant differences in abundance, “caries-specific” and “caries-free-specific” taxa (present in the caries or the caries-free population but absent in the other) were found (Figure S5). *H. paraphrohaemolyticus HK411, Neisseria sicca 4320, Neisseria* sp. *oral clone AP132, Actinobacillus pleuropneumoniae MCCM 00189*, and *Streptococcus* sp. *oral clone ASCE06* were found in caries-free children but were absent in the children with caries, which suggests that these bacteria might be crucial in supporting a healthy oral ecosystem. The caries-specific taxa, including *Lactobacillus* spp., *Prevotella* spp., *Streptococcus* spp., and *Veillonella* spp., etc., may play an important role in the occurrence and development of caries (Figure S5).

From Figure S5, we can see that *Lactobacillus* sp. *C56* was found in half of the children with caries but was absent in the caries-free children. *Streptococcus mutans* was found in nearly 80% of the children with caries, while it was only found in one caries-free child. The distribution of *D. pneumosintes* in children with caries and caries-free children was 50 and 14.3%, respectively. *Mogibacterium pumilum*, which has been isolated from the periodontal pockets of patients with periodontal disease and infected root canals (Nakazawa et al., 2000), was detected in the saliva samples of 11 children with caries. The detected rate in the children with caries and the caries-free children was 55 and 14.3%, respectively.

*Streptococcus rubneri*, which was first obtained from throat samples of healthy humans (Huch et al., 2013), was found in the saliva samples of 14 caries-free children but was only found in samples from 2 of the caries children. This difference suggests the importance of this bacterium in maintaining a healthy oral micro-ecological environment.

### The core oral microbiota in children with caries and caries-free children

**Figure 6:**
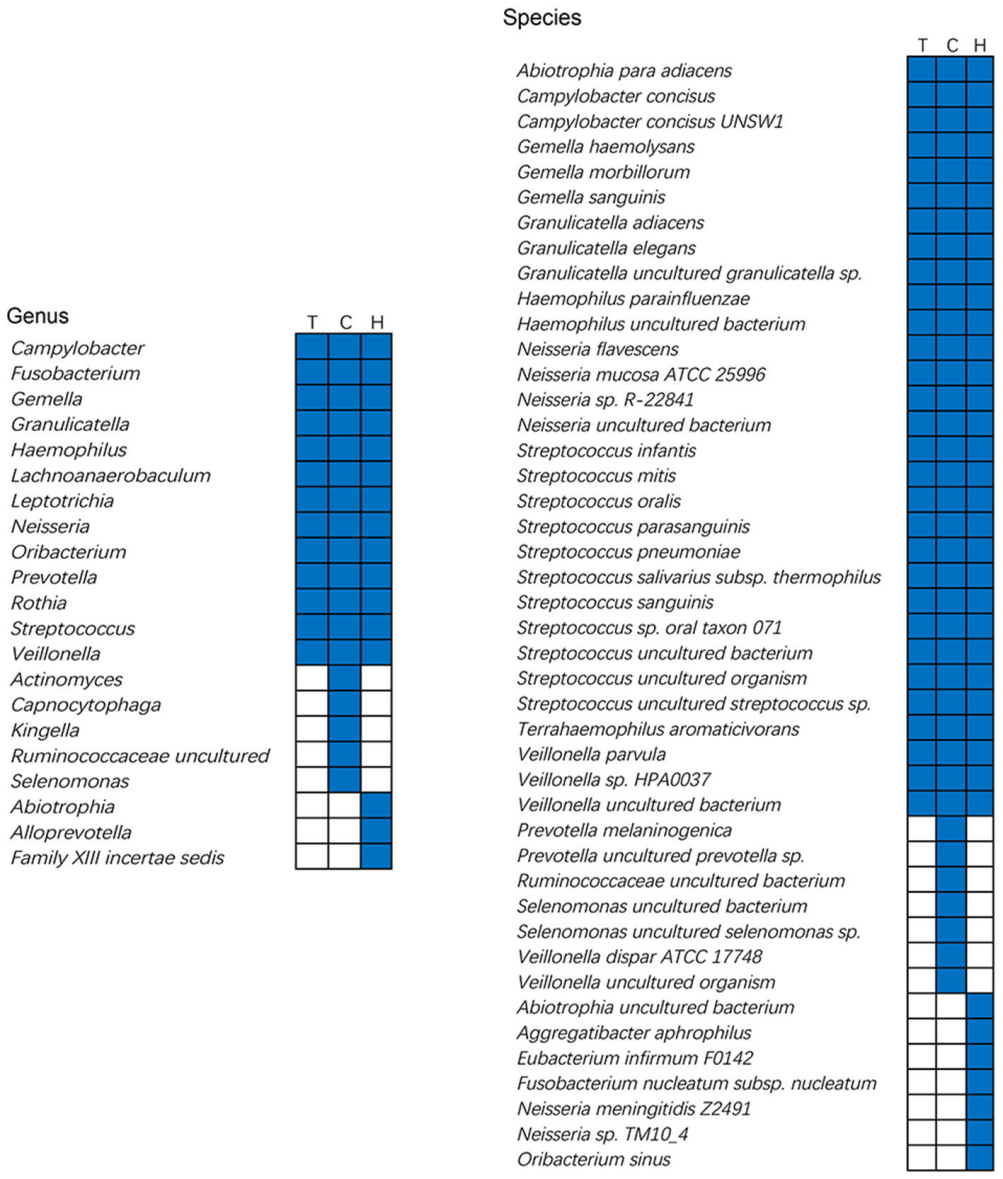
Core microbiome of caries and caries free group at the genus and species level. Each column represents a group. For each column, the black cells indicate that the species or genus listed on the left has fallen into the “core microbiome” of this group and white cells indicate that the species or genus listed on the left could not be observed in all the samples of this group. T, total subjects; C, Caries children; H, Caries free children.

The core microbiota of all subjects at the genus and species levels was included in Figure 6. Core taxa associated with the caries and caries-free groups were compared. Ultimately, 13 core taxa from 110 genera were present across all individuals; 18 core taxa from 100 genera were found in the caries group; and 16 core taxa from 80 genera were present in the caries-free group. At the species level, 31 core taxa from 876 species were present across all individuals; 37 core taxa from 743 species were observed in the caries group; and 39 core taxa from 694 species were present in the caries-free group.

To better interpret the core microbiota, we calculated the percentage of the core taxa according to the genus and species abundance of the entire community. The bacteria that showed 100% frequency in all subjects were defined as the whole core microbiota (W-core microbiota), the bacteria present in 95% of all subjects were defined as the 95% core microbiota, etc. (Table S5). As Table S5 shows, the abundance of the W-core microbiota in the caries-free group was higher than in the caries group at both the genus and species levels. The same trends were also found for the 95, 90, and 85% core microbiotas at different taxonomic levels. We speculate that the structure of the core microbiota in caries-free children is more stable than that in the children with caries for different bacterial taxonomic profiles.

### Deep analysis based on grouping with caries developing for 6 month follow-up

The oral condition of the children in both groups was followed up 6 months after sample collection. According to the results of the follow-up oral examinations, 16 subjects in the caries-free group (21 subjects) maintained a caries-free state, with dmfs remaining at zero; however, development of new dental caries was observed in 5 children (Table S2). Thus, the 41 subjects were divided into a caries group (C group), a transitional group (H-C group), and a caries-free group (H group) based on the follow-up results.

**Figure 7:**
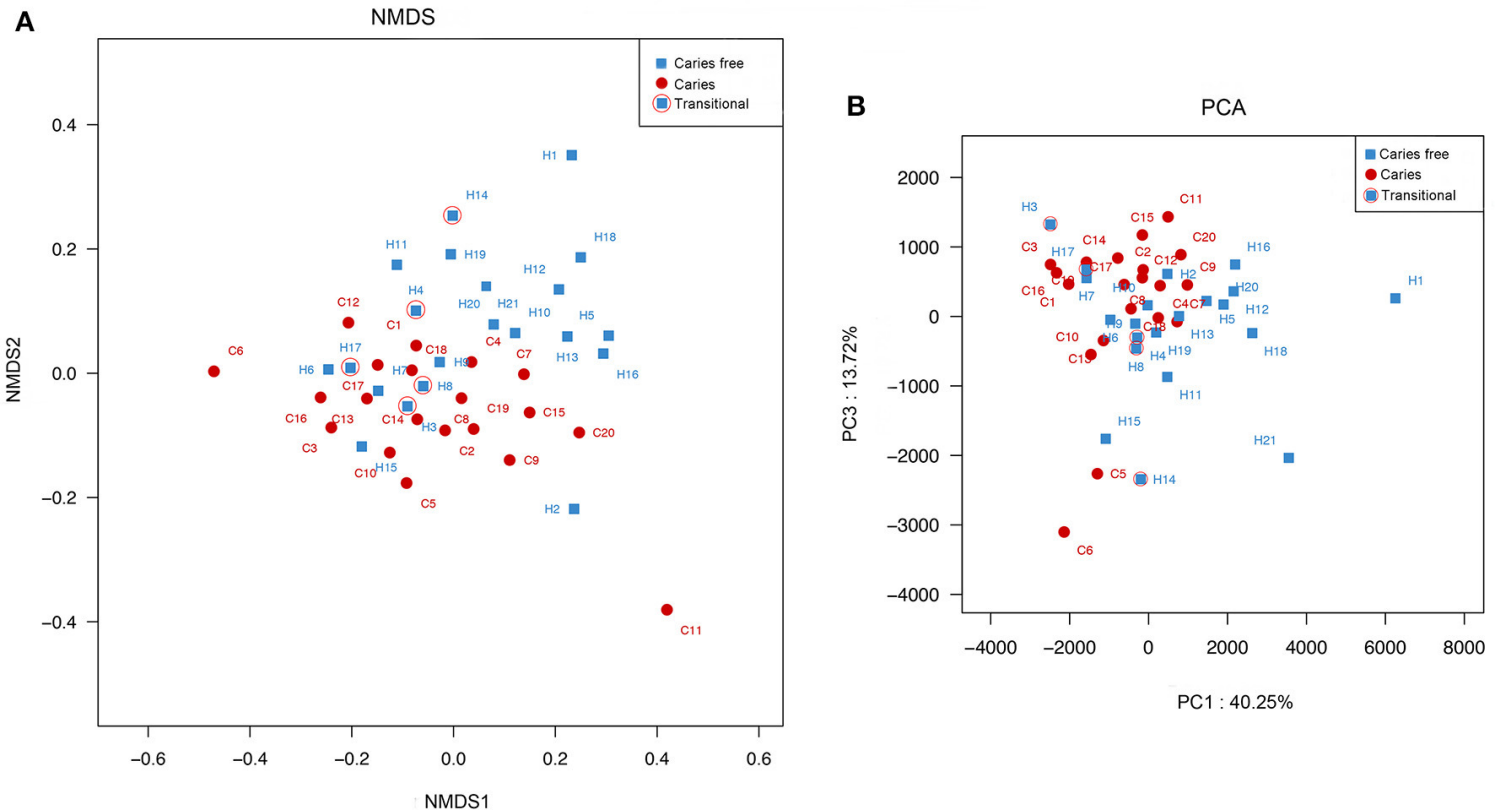
**(A)** Principle Component Analysis among caries and caries free children was based on euclidean metric distance. **(B)** Principal co-ordinates analysis between the two groups was based on other similarity measures. Each symbol represented a sample.

To assess the microbial community structure alteration in the microbiota associated with caries, all the children were clustered via principal component analysis (PCA) based on the relative abundance of OTUs (Figure 7). The caries and caries-free groups displayed structural shifts, along with a considerable overlap (Figure 7), indicating that there should be a transition state between being caries-free and having caries. This phenomenon showed that many caries-free children might eventually suffer from dental caries, which was proven in the follow-up examination after 6 months (Table S2). Similarly, the shifts of the oral microbiota between the two groups were evaluated using nonmetric multidimensional scaling (n-MDS) ordination based on a beta diversity distance measure (Figure 7).

### Taxonomic analysis and comparison among children in the caries, caries-free, and transitional groups

**Figure 8:**
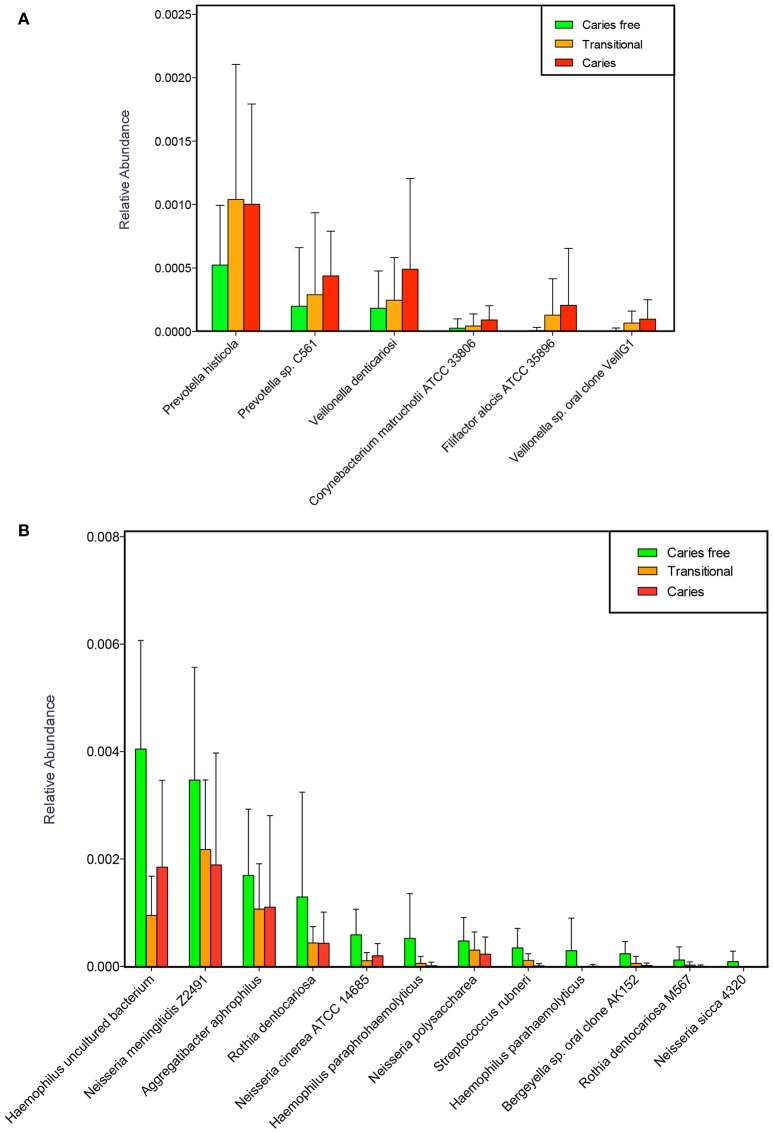
Species that up-regulated **(A)** and down-regulated **(B)** in the progression of dental caries.

The relative abundance of all species was further compared, and the changes in the transitional oral microbiota during the development of dental caries were then analyzed. Compared with the caries-free children, 6 species, including *P. histicola, Prevotella* sp. *C561, V. denticariosi, Corynebacterium matruchotii ATCC 33806, Filifactor alocis ATCC 35896*, and *Veillonella* sp. *oral clone VeillG1*, showed elevated abundance in the transitional and caries groups(Figure 8A). *Haemophilus* spp., *Neisseria* spp., *Rothia* spp., *A. aphrophilus, Bergeyella* sp. *oral clone AK152*, and *S. rubneri*, which were more abundant in the caries-free group, exhibited a relatively low abundance in both the caries group and the transitional group after the 6 month follow-up (Figure 8B).

## Discussion

Elucidating and analysing the genetic diversity of complex microbial populations requires reliable and sensitive sequencing techniques. PacBio RS II sequencing, which has the ability to provide longer sequences and sequencing reads, was used on the entire 16S rRNA gene. Compared to earlier sequencing techniques, this platform can establish a higher estimate of richness and provide the ability to identify organisms at a higher resolution (Mosher et al., 2014).

In our study, a total of 702,304 high-quality sequences were produced, and 877 species were analyzed, which suggests that this approach is useful for estimating the details of the oral microbiota at a more in-depth level. In previous studies, many researchers focused on the relationship between microorganisms and the development of caries; however, most of the cariogenic bacteria were only identified at the genus level (Jiang et al., 2013, 2014). Species-level and even strain-level resolution is thought to be important for caries prognosis (Yang et al., 2012). To the best of our knowledge, this study is the first to characterize the oral microbiota in childhood caries at the species level based on single-molecule real time sequencing.

In this study, the OTU number, Chao and ACE index were all identical in each sample. The reason is that according to the formula of richness indices, none of the 3287 OTUs has only one reads and the value of n1 is zero. From the richness indices, a slightly higher species richness was found in the children with caries, but there was no statistical significance.

Discovering the core microbiota is important for understanding the stable components across complex microbial assemblages. The core microbiota has been defined as a set of microbial organismal lineages that are shared in a given microecosystem across all or the majority of microbiotas (Hong et al., 2015; Xu et al., 2015). Our previous study has indicated that there might be a core microbiota in the oral cavity, and some bacteria have been found in all subjects at the genus level (Jiang et al., 2013). From Figure 6, we can see that *Abiotrophia* spp. and *Neisseria* spp., among others, might be associated with a healthy oral microbial ecosystem, whereas *Prevotella* spp. and *Veillonella* spp. may be the risk factors related to dental caries. Compared with a previous study on core microbiome in 37 adolescents, 53.8% of the core genera detected in this study including *Campylobacter, Fusobacterium, Granulicatella, Lachnoanaerobaculum, Prevotella, Streptococcus*, and *Veillonella* are consistent. However, only 4 species including *Granulicatella adiacens, S. mitis, Streptococcus sanguinis*, and *Veillonella parvula* were consistent with previous results (Johansson et al., 2016). Therefore, we speculated that a core microbiome is linked to age, races and regions at the species level. A recent study by Xuedong Zhou and colleagues has also demonstrated that the core oral microbiota is better defined based on age and oral niches (Xu et al., 2015). Here, we compared the core microbiota of Chinese children with or without caries at the genus and species levels, respectively. The analysis of core species in caries children has greatly enriched and deepened the concept about core microbiota in disease states. A shift in the oral core microbiota was observed in the two groups, which strongly supports our previous study.

Of the 14 bacterial phyla covered by the Human Oral Microbiome Database (HOMD), 13 were detected in our research. From Figure 3A, we can see that two groups of bacteria are dominant in the oral cavity, the Firmicutes and the Proteobacteria. Here, we show that the relative proportion of Proteobacteria is decreased in children with caries in comparison to the group of caries-free children. Our findings indicate that dental caries have a microbial component, which might have potential therapeutic implications. Our previous research, which analyzed dental plaque in children with severe ECC, showed that children with caries had fewer Proteobacteria and more Firmicutes than did the caries-free controls, which is consistent with the results of this study (Chen and Jiang, 2014).

At the genus level, the abundance of *Rothia* in this study was lower in the children with caries (*p* < 0.05), which is consistent with the trends of our previous data by 454 sequencing (Chen and Jiang, 2014) and unpublished data using metagenomics sequencing methods, although this result was not statistically significant (*p* > 0.05). However, Jagathrakshakan and Thomas' sequencing analysis indicated a higher prevalence of *Rothia* in a group with caries compared to controls (Jagathrakshakan et al., 2015). The cause of these inconsistent findings might be the difference between research methods and the different ages, races and origins of the enrolled subjects. These conflicting results require further study.

At the species or strain level, the acidogenic-aciduric bacterial species *Lactobacillus* was generally accepted as the leading candidate in the causation of caries prior to the 1950s, before *S. mutans* began to dominate the literature in 2008 (Badet and Thebaud, 2008). In previous investigations, *Lactobacillus* was highly prevalent in the caries group but almost absent from caries-free children (Gross et al., 2010; Shimada et al., 2015). In this study, *Lactobacillus* spp., including *L. fermentum, L. gasseri, L. mucosae, Lactobacillus* sp. *C56*, and *Lactobacillus vaginalis*, were found in more than 20% of the children with caries. In the caries-free group, however, *Lactobacillus* spp. had a low prevalence, and the above five species were not found (Figure S5). However, it was hypothesized that *Lactobacillus* must rely on other acid producers, such as *S. mutans*, to initiate caries because of its weak adhesive force (Jiang et al., 2014).

Among the hundreds of oral bacterial species *S. mutans* is assumed to be the species most strongly associated with human dental caries. It is recognized to be the primary pathogen in caries because of its ability to form a rigid biofilm on tooth surface and to continuously produce acid (Marchant et al., 2001). In this study, *S. mutans* was abundant in children with caries, being found in nearly 80% of them, while it was only found in one of the caries-free children. Our research indicated that *S. mutans* appeared to be prevalent and strongly associated with caries, which was consistent with previous studies. This finding was expected, since it is well documented that *S. mutans* has a high capacity to adhere to enamel, produces lactic acid and contributes to caries pathogenesis. Higher prevalence of *S. mutans* has frequently been associated with cariogenesis, though not all children with caries test positive for *S. mutans*. Interestingly, according to the results of the follow-up oral examinations, the caries-free child with *S. mutans* detected maintained a caries-free state 6 month later. This finding suggested us that although *S. mutans* has been recognized as cariogenic bacteria, it also can be detected in oral samples from caries-free children, which suggested that caries is an outcome of the overall activity of a heterogeneous mixture of microorganisms. *Streptococcus mutans* is not the only cariogenic bacteria and other bacterial species might also be responsible for caries initiation and development. What's more, interactions between bacteria in multispecies biofilms can affect the cariogenic ability of *S. mutans*. While *S. mutans* is known to occur with higher prevalence, several other acidogenic species have also been reported to co-prevail within niche areas, such as supra- and sub-gingival plaque substances and dentinal caries (Jagathrakshakan et al., 2015).

*Dialister* was isolated from the root canals of patients with endodontic infections and a deep periodontal pocket by Downes in 2003 (Downes et al., 2003). The presence of this species was thought to be a factor of chronic apical abscesses (Tennert et al., 2014) and peri-implantitis (da Silva et al., 2014); however, the association between *D. pneumosintes* and caries had not previously been identified.

The commensal organisms *Neisseria* spp., including *N. sicca 4320* and *Neisseria* sp. *oral clone AP132*, were found only in the caries-free children, which has not been reported previously. Similarly, *H. paraphrohaemolyticus HK411* was found in 1/3 of the caries-free children but was absent in all of the children with caries. From the results, we speculated that the above strains may play an important role in the balance of the oral microbiota as antagonistic cariogenic bacteria strains. Besides, to our knowledge, a correlation between *P. histicola, Selenomonas flueggei, M. pumilum*, and *Mogibacterium timidum* and caries has not been previously reported.

Based on the results of 6 months follow up, we speculate that the species elevated in the transitional group including *Prevotella* spp., *Veillonella* spp., *Corynebacterium* spp., and *Filifactor* spp. might be the caries-associated microbes in children. In addition, among them, *P. histicola*, which was first isolated from the human oral cavity in 2008 (Downes et al., 2008), exhibited a relatively high abundance in the transitional group, even higher than in the caries group. This result suggests that *P. histicola* might play an important role in the pathogenesis of dental caries. However, *Veillonella* species probably do not directly contribute to caries. They utilize lactic acid for growth and therefore benefit from the acidic environment within carious lesions (Delwiche et al., 1985; Do et al., 2015). *Haemophilus* spp., *Neisseria* spp., *Rothia* spp., and *Streptococcus* spp., which exhibited a relatively low abundance in the transitional group might be associated with maintaining the status of being caries-free. However, a more large-scale investigation is needed to further confirm which microbes play an important role in maintaining the status of a caries-free micro-ecological environment and the microbes that play a key role in initiating caries.

Some recognized caries pathogens associated with severe ECC, such as *Scardovia wiggsiae* (Tanner et al., 2011a,b), were not significantly different between the two groups in this study. The possible reasons partly because the samples were collected from populations of different regions and from different oral nichs and partly because different methods were applied. Also, we should note that the sequencing data generated in this study showing only an association between oral microbiota and dental caries. More verification research is clearly needed to determine the cariogenic mechanism of certain microorganisms.

In this research, we demonstrated the diversity and complexity of the microbial community associated with caries at the species or strain level and were able to infer that after the first colonization of *S. mutans* on a tooth surface, other species such as *Lactobacillus, Prevotella* spp., and *Veillonella* spp. then stick to the tooth surface. Subsequently, various organic acids are produced, and dental caries occurs. Other bacterial species, such as *D. pneumosintes*, also be associated with caries. According to the results of follow-up oral examinations, the pivotal species in the progression of dental caries were discovered; however, little is known about the mechanisms underlying the process of caries development. Thus, further work is needed to reveal the pathogenic mechanism and signal transduction pathways associated with oral cariogenic bacteria in the process of dental caries.

## Conclusions

1. Changes in the abundance of certain taxa, such as an overabundance of *Lactobacillus* spp., *Prevotella* spp., and *Veillonella* spp. species, distinguished microbiota associated with caries from caries-free microbiota; furthermore, children with and without caries possessed different arrays of *Streptococcus* species.
2. Dental caries is not dominated by one particular bacterium but is, in fact, a complex community formed by tens of bacterial species. The core microbiota in children with and without caries have shown that the caries-free oral micro-ecological niche is more stable.
3. A certain group of bacteria plays a considerable role in the process of dental caries pathogenesis. “Caries-specific” and “caries-free-specific” taxa were detected, and the pivotal species in the progression of dental caries at the species level were discovered.
4. Caries microbiotas were slightly more variable in oral community structure, whereas the caries-free groups were relatively conserved.
5. Over-represented and under-represented species were observed in the transitional state from caries-free to caries-containing microbiotas.
6. Compared to traditional 16S rRNA sequencing technology, the third-generation sequencing PacBio RS II system improved read lengths and annotation of the nucleotide sequences of oral bacteria to the strain level.

## Data accessibility

The datasets used and/or analyzed during the current study available from the corresponding author on reasonable request.

Sequence files and metadata for all samples used in this study have been deposited in SRA (PRJNA388240; SRP108162). The number of sequences obtained and children Metadata which includes sample ID, gender, age, weight, dmft, and dmfs. have all been included as additional files Tables S2, S3, respectively.

## Author contributions

HC, YuW, JZ, XC, WJ, YiW, PZ, YT, and XL carried out experiments; WJ, SW, LX, and JZ provided technical and intellectual support; YuW, JZ, and HC analyzed experimental data and participated in writing the manuscript; YuW and HC participated in organizing manuscript; HC conceived the project, designed the experiments, organized, wrote, revised and finalized the manuscript with the help of all authors.

### Conflict of interest statement

The authors declare that the research was conducted in the absence of any commercial or financial relationships that could be construed as a potential conflict of interest.

## Supplementary material

Figure S1 OTU distribution between caries and caries free children from 41 oral samples.

Figure S2 Comparison of α-diversity and coverage between caries and caries free children. **(A)** Shannon index, which can reflect how evenly the OTUs are distributed among oral microbiome; **(B)** Simpson index, which is to measure the degree of concentration when oral microbime are classified into OTUs; **(C)** Coverage, which is calculated from the length of the original genome (G), the number of reads(N), and the average read length(L) as N^*^L/G.

Figure S3 Richness of oral saliva from 41 samples in our research. **(A)** OTU number; **(B)** Ace index; **(C)** Chao index.

Figure S4 Relative abundance of bacteria in the caries and caries free children. **(A)** Top10 phylum in caries group; **(B)** Top10 phylum in caries free group; **(C)** Top10 genera in caries group; **(D)** Top10 genera in caries free group; **(E)** Top10 species in caries group; **(F)** Top10 species in caries free group.

Figure S5 **(A)** The distribution of the species that enriched in caries children in the 41 samples in our research. ^*^Species exist only in caries children; **(B)** The distribution of the species that enriched in caries free children in the 41 samples in our research. ^*^Species exist only in caries free children.
