# Supplementary figures and images for: Profiling of Oral Microbiota in Early Childhood Caries Using Single-Molecule Real-Time Sequencing
Source: Front Microbiol. 2017 Nov 15;8:2244. doi: 10.3389/fmicb.2017.02244 (PMC5694851; doi:10.3389/fmicb.2017.02244)

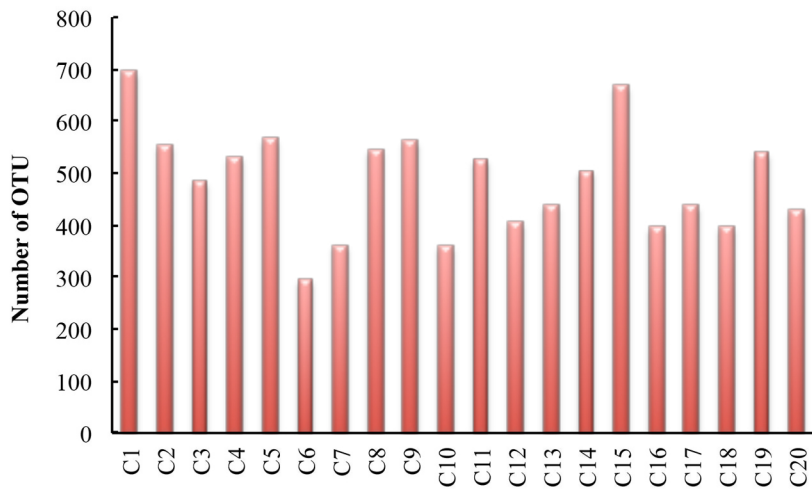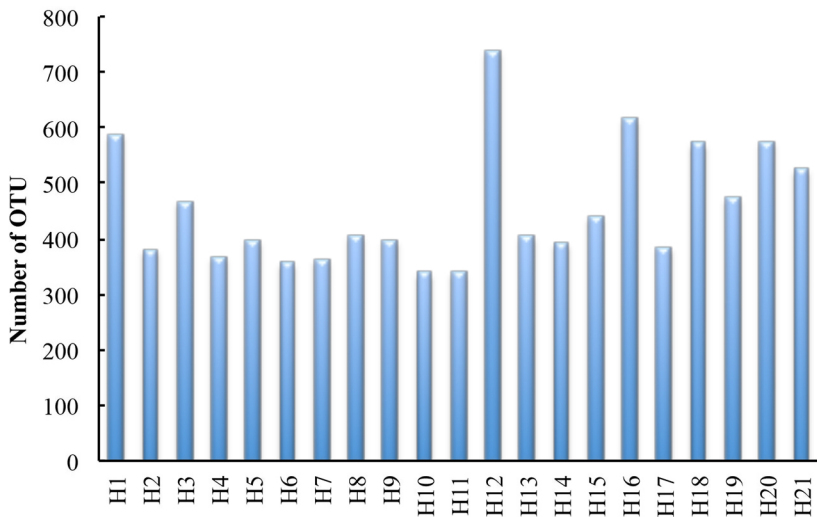

Supplement: Figure S1 — OTU distribution between caries and caries free children from 41 oral samples. [file Image1.PDF]

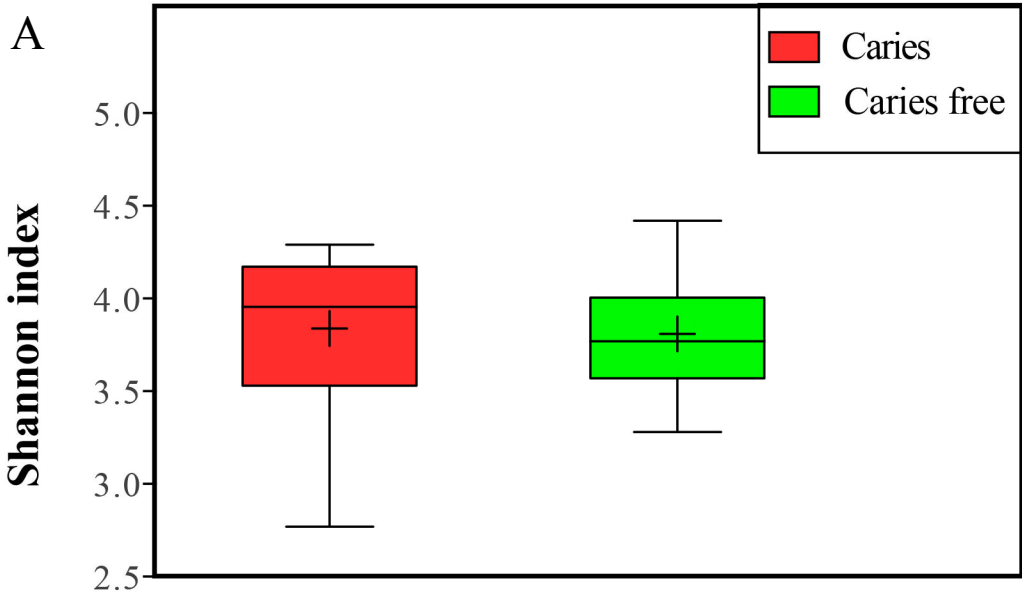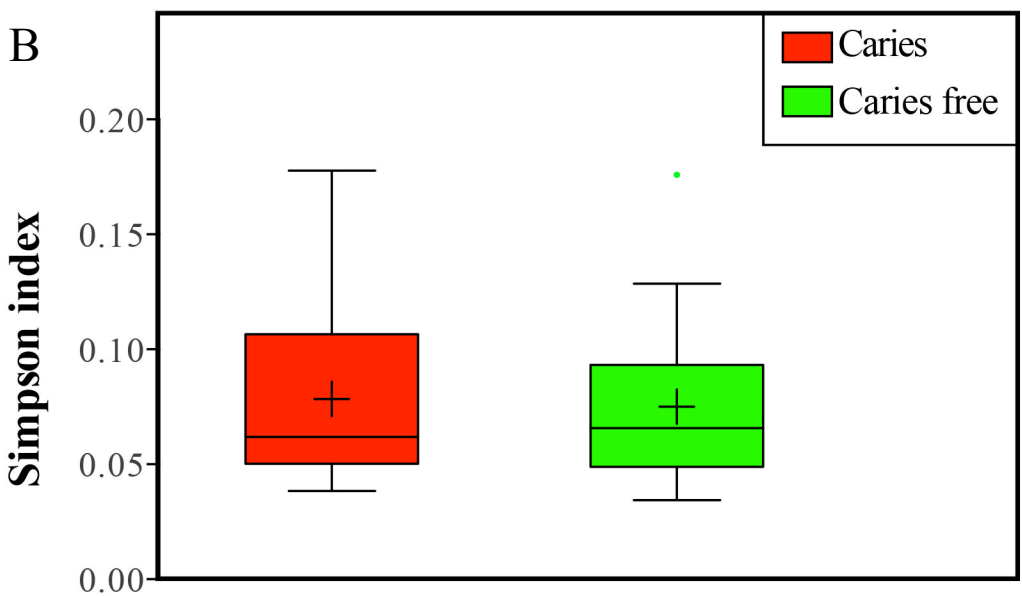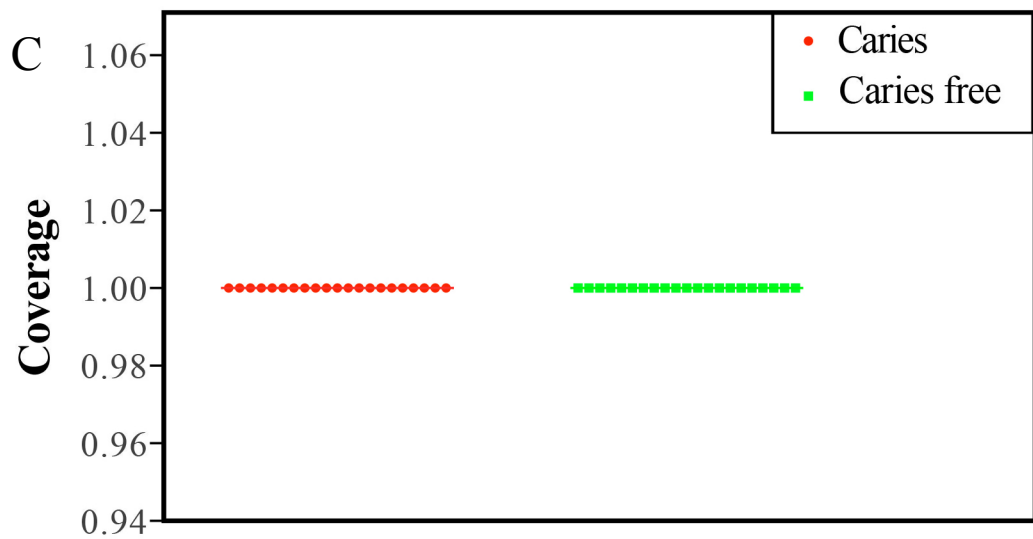

Supplement: Figure S2 — Comparison of α-diversity and coverage between caries and caries free children. (A) Shannon index, which can reflect how evenly the OTUs are distributed among oral microbiome; (B) Simpson index, which is to measure the degree of concentration when oral microbime are classified into OTUs; (C) Coverage, which is calculated from the length of the original genome (G), the number of reads(N), and the average read length(L) as N*L/G. [file Image2.pdf]

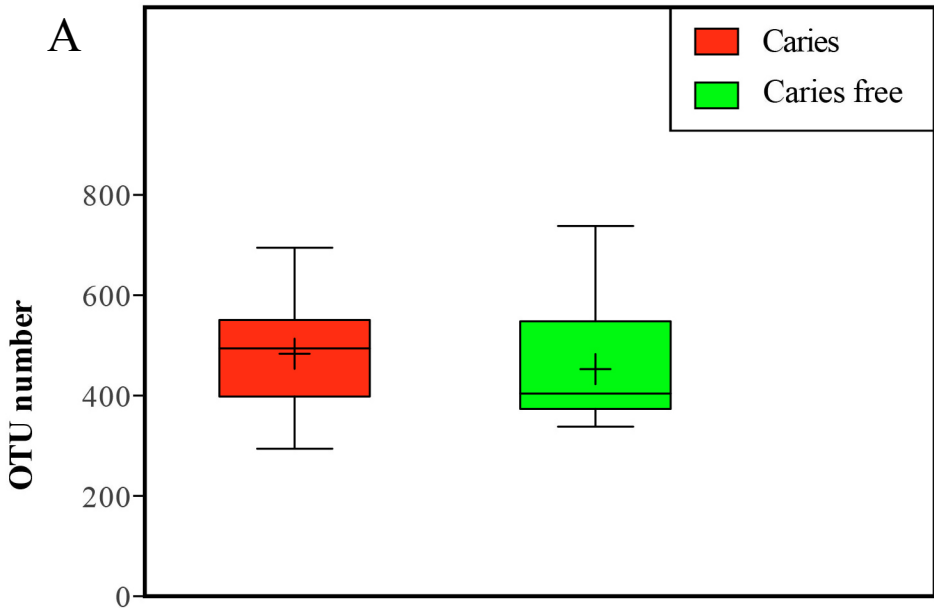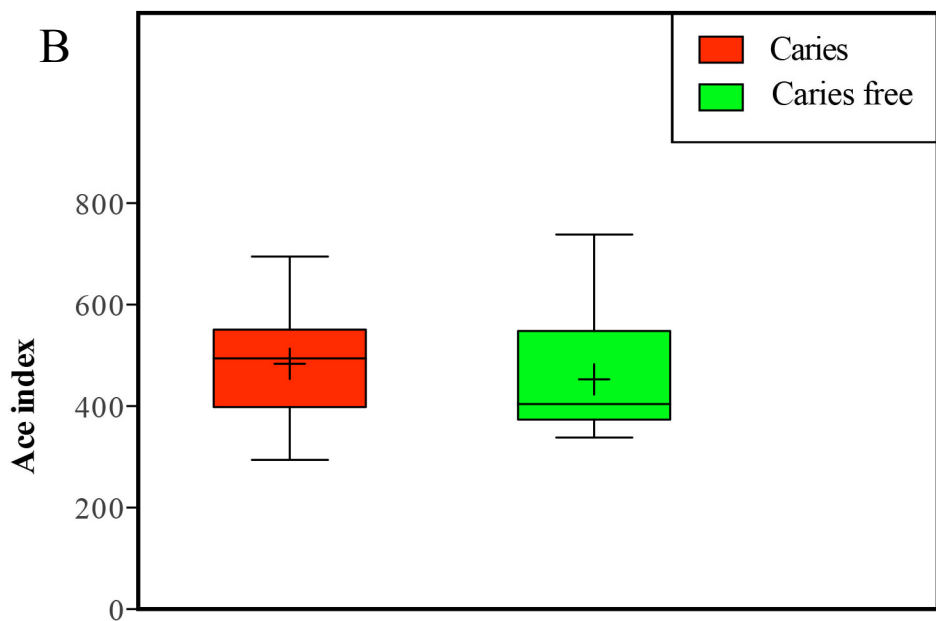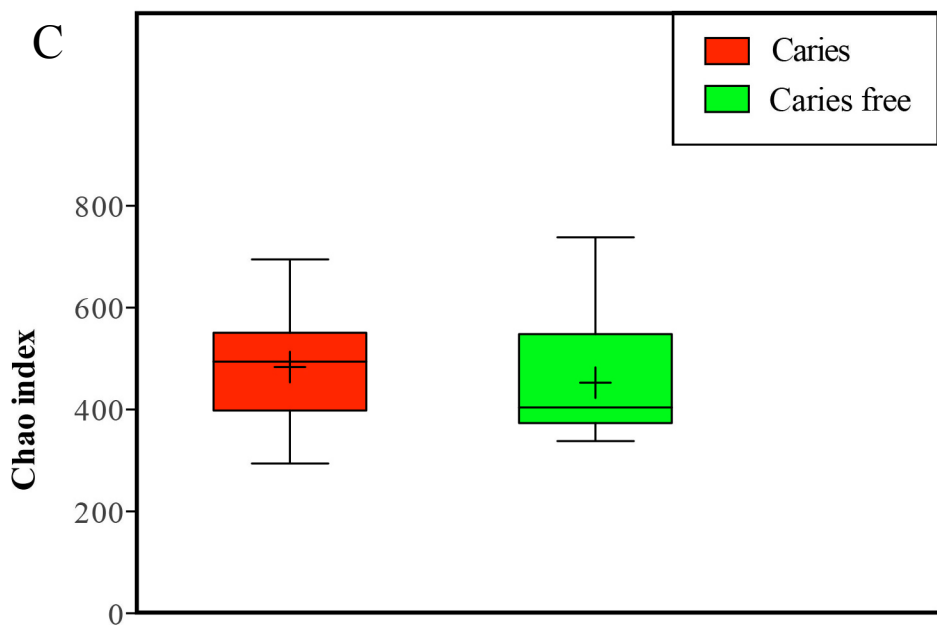

Supplement: Figure S3 — Richness of oral saliva from 41 samples in our research. (A) OTU number; (B) Ace index; (C) Chao index. [file Image3.pdf]

## Phylum

**A**

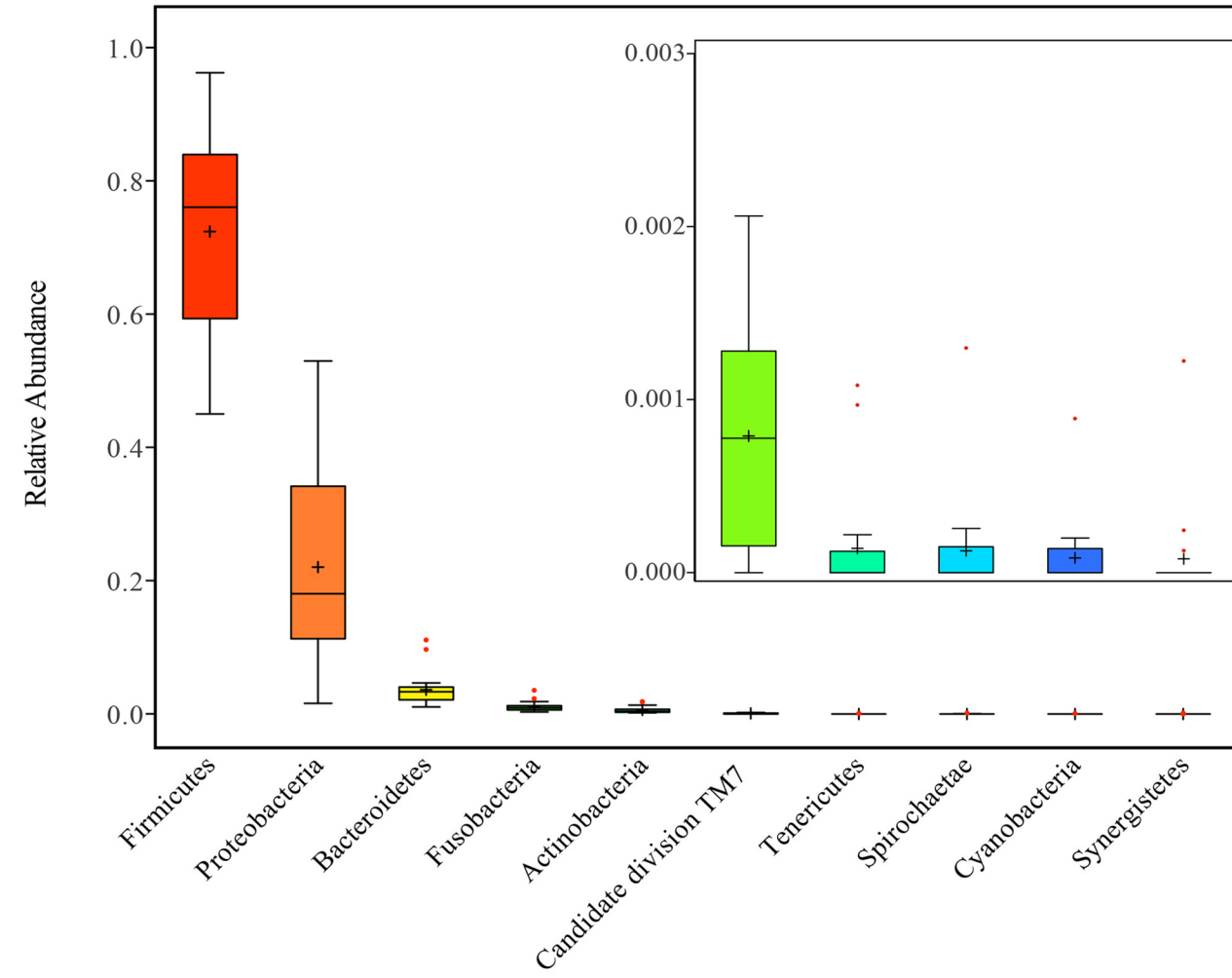

## Phylum

**B**

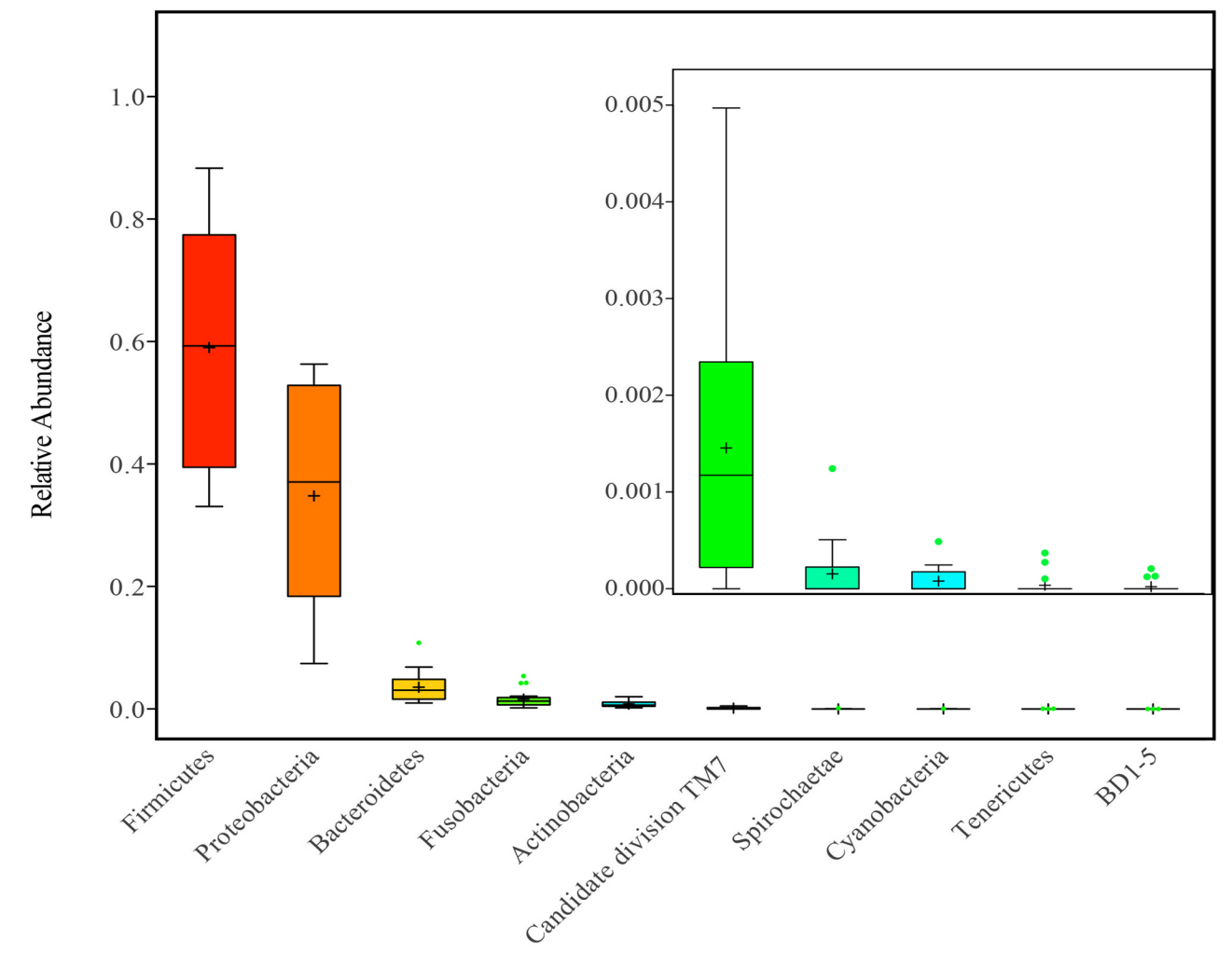

## Genus

**C**

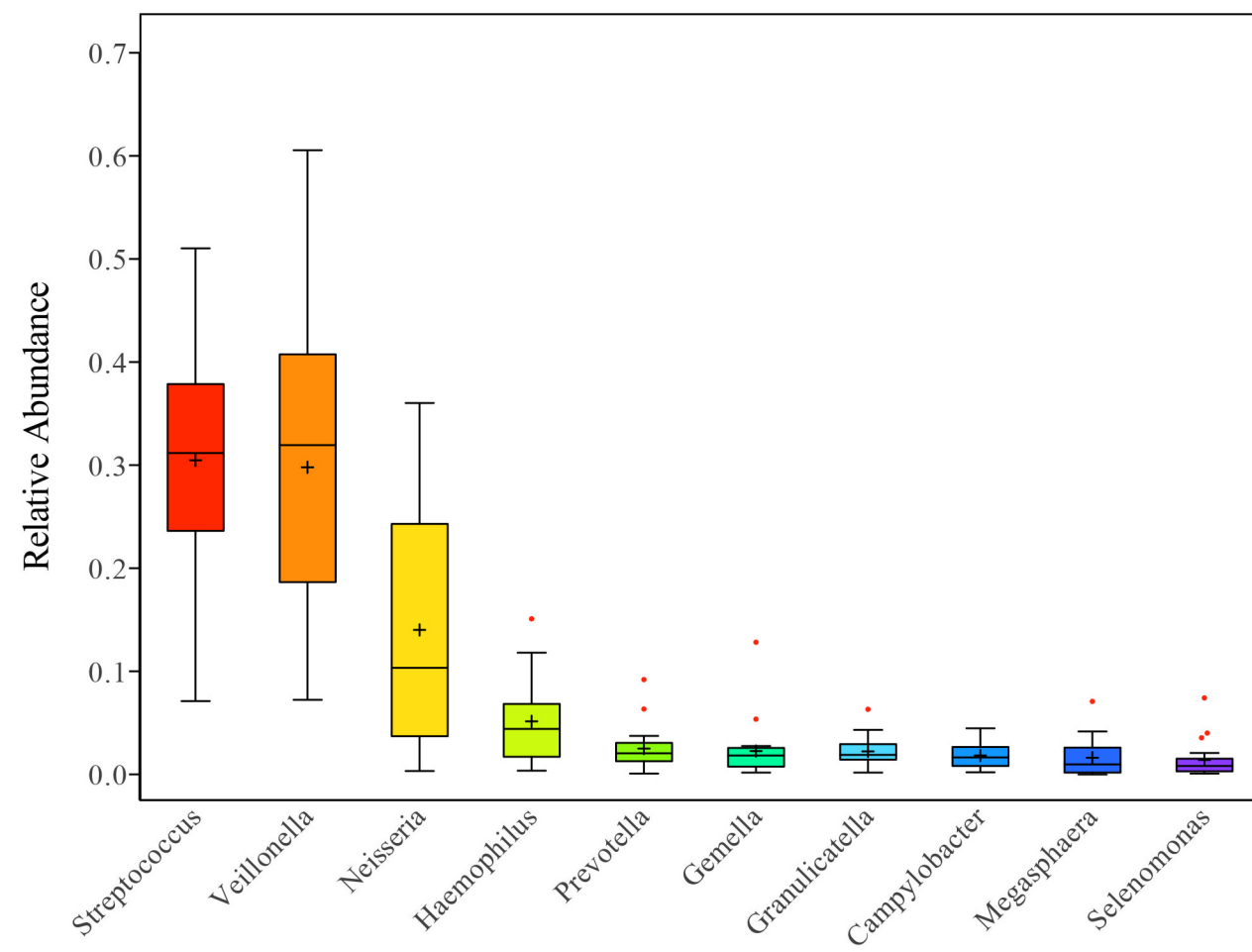

## Genus

**D**

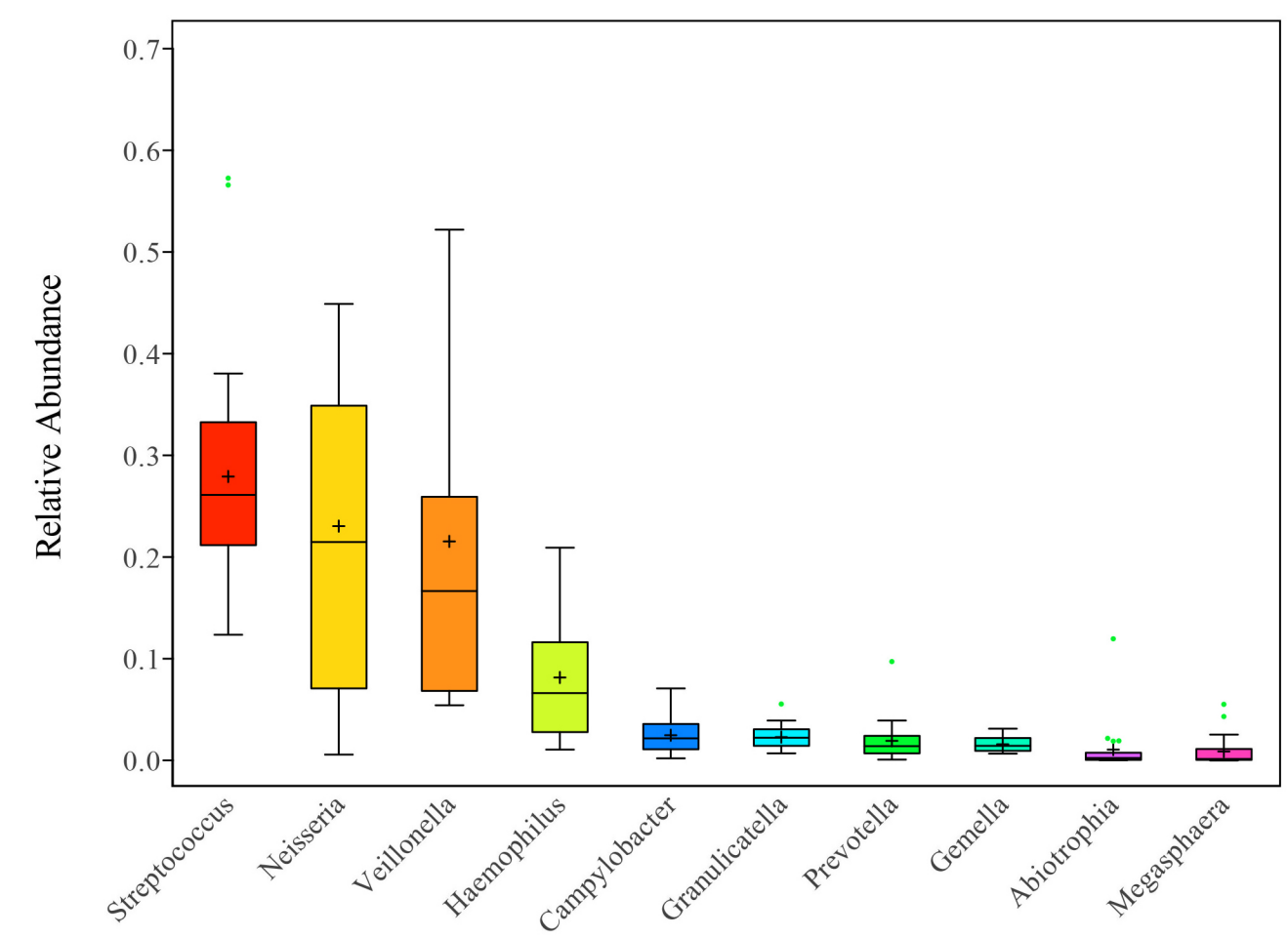

## Species

**E**

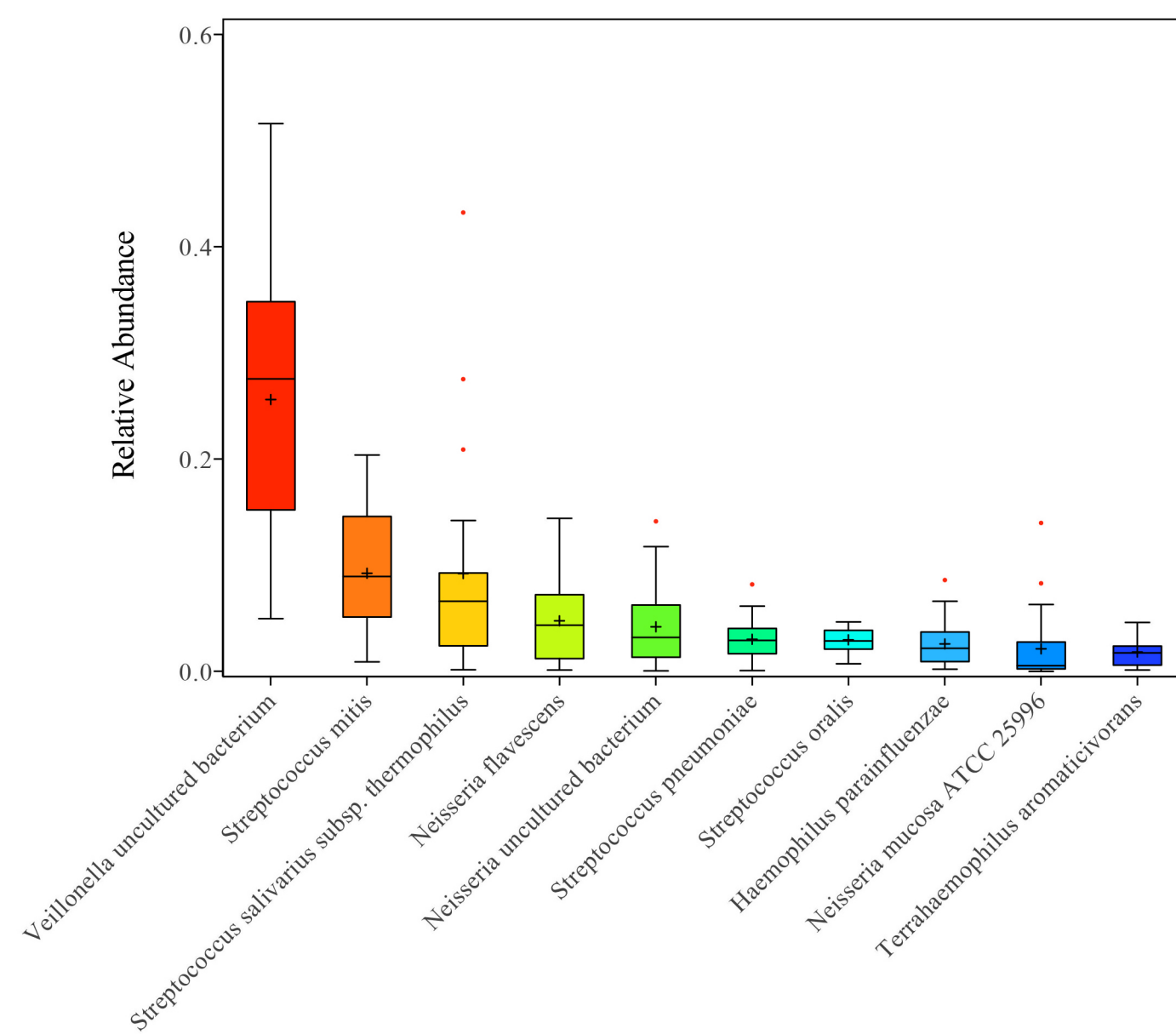

## Species

**F**

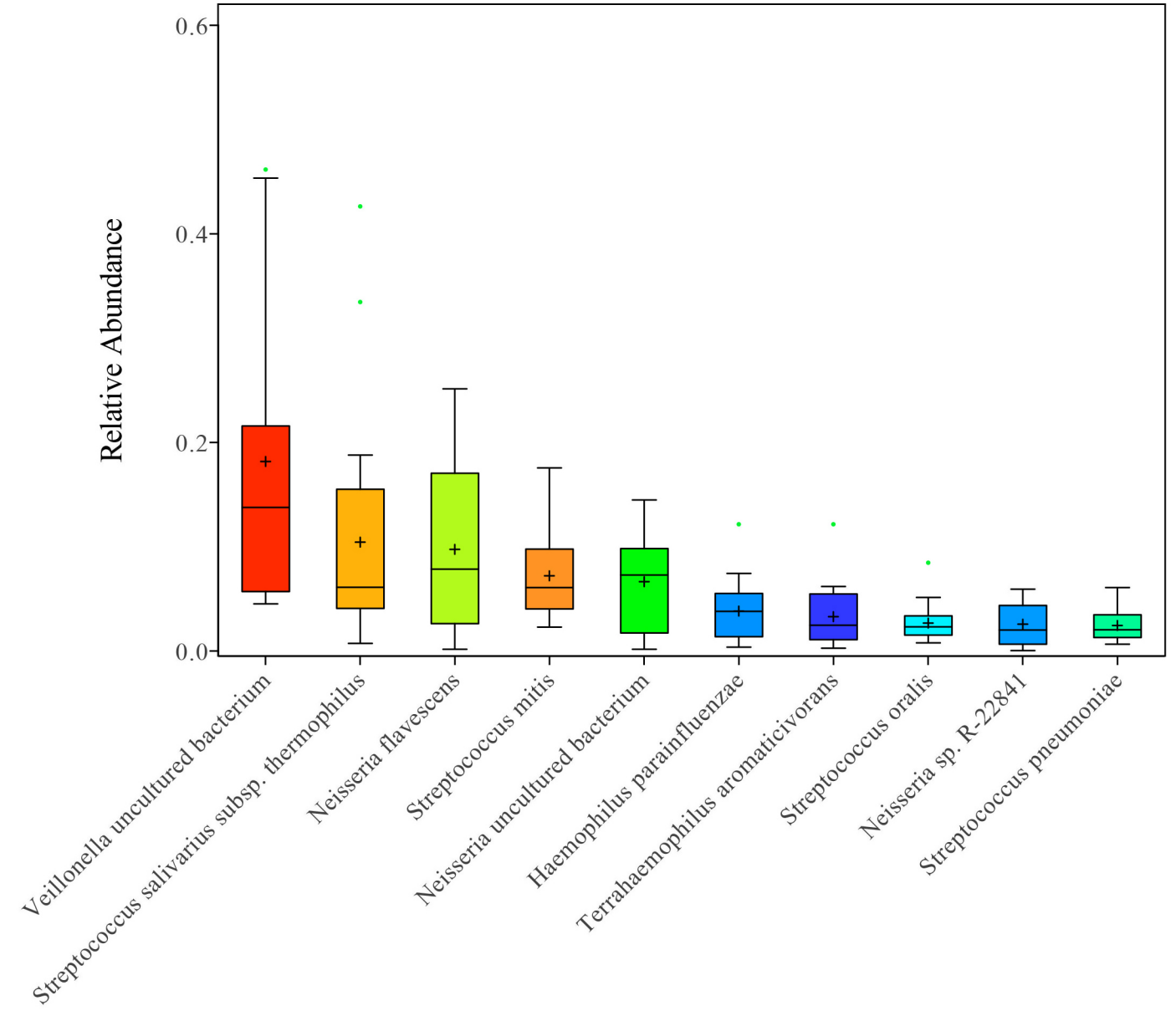

Supplement: Figure S4 — Relative abundance of bacteria in the caries and caries free children. (A) Top10 phylum in caries group; (B) Top10 phylum in caries free group; (C) Top10 genera in caries group; (D) Top10 genera in caries free group; (E) Top10 species in caries group; (F) Top10 species in caries free group. [file Image4.PDF]

A

..... Caries      ..... Caries free

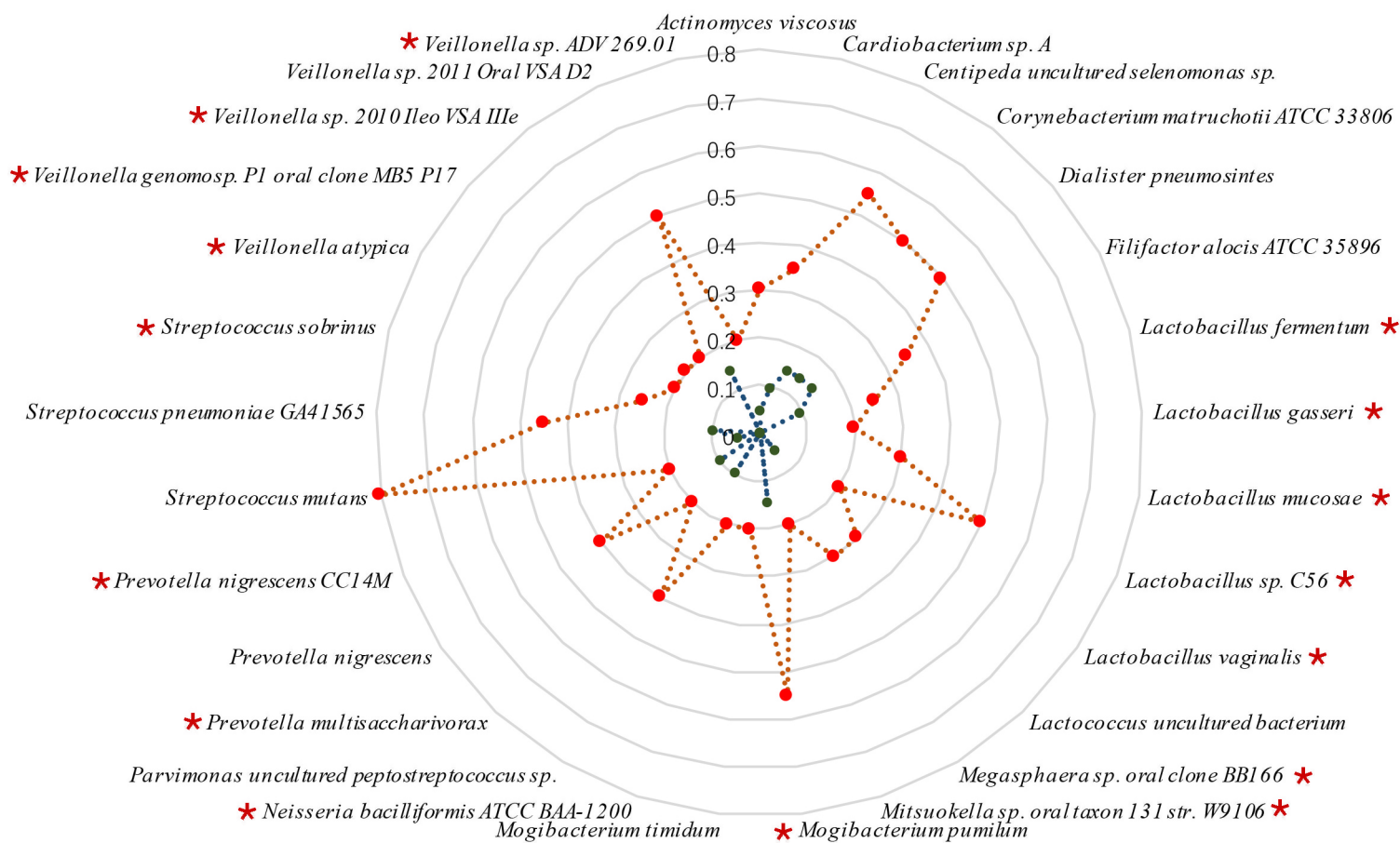

B

..... Caries      ..... Caries free

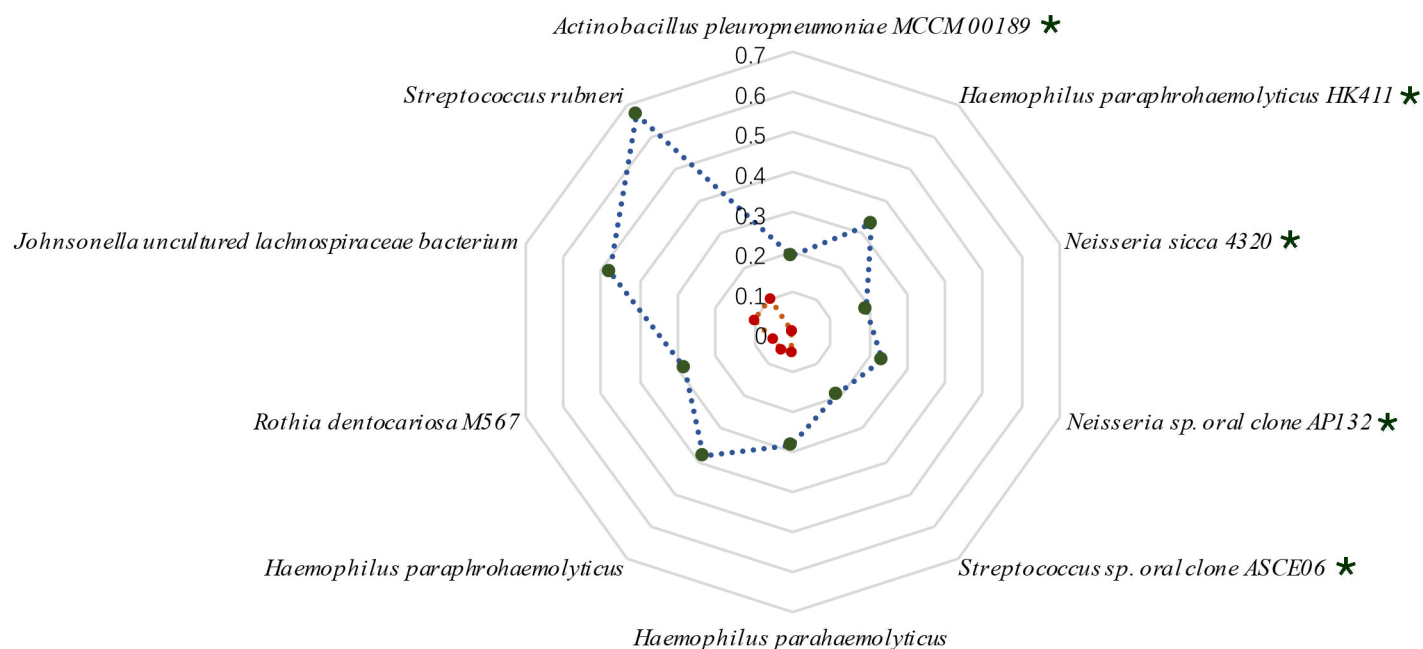

Supplement: Figure S5 — (A) The distribution of the species that enriched in caries children in the 41 samples in our research. *Species exist only in caries children; (B) The distribution of the species that enriched in caries free children in the 41 samples in our research. *Species exist only in caries free children. [file Image5.PDF]
