# Supplementary material for: Profiling of Oral Microbiota in Early Childhood Caries Using Single-Molecule Real-Time Sequencing
Source: Front Microbiol. 2017 Nov 15;8:2244. doi: 10.3389/fmicb.2017.02244 (PMC5694851; doi:10.3389/fmicb.2017.02244)
Supplement: Supplementary file 6 [file Table1.PDF]

Table S1. Phenotype information of the Chinese children in research (41 samples).

Characteristics	Age* (months)	Gender (male/female)	dmft*	dmfs*
Caries children (n=21)	64.0±5.74	12 / 9	12.3±1.90	20.5±10.58
Caries free children controls(n=20)	64.8±6.13	10 / 10	0	0

dmft:the total number of teeth that are decayed, missing or filled

dmfs: the total number of surfaces that are decayed, missing or filled

*Data are expressed as the mean±standard deviation.
