# Supplementary material for: Profiling of Oral Microbiota in Early Childhood Caries Using Single-Molecule Real-Time Sequencing
Source: Front Microbiol. 2017 Nov 15;8:2244. doi: 10.3389/fmicb.2017.02244 (PMC5694851; doi:10.3389/fmicb.2017.02244)
Supplement: Supplementary file 7 [file Table2.PDF]

**Table S2**. Characteristics of caries and caries-free children.

| Group | Sample ID | Country | Gender | Age (months) | Weight（kg） | Height（cm） | dmft | dmfs | dmft (6 month later) | dmfs (6 month later) | antibiotics in 3 months（Y or N） | fluoride treatment in 3 months（Y or N） |
|---|---|---|---|---|---|---|---|---|---|---|---|---|
| | H1 | China | Female | 73 | 19.5 | 114.5 | 0 | 0 | 0 | 0 | N | N |
| | H2 | China | Male | 60 | 17 | 109 | 0 | 0 | 0 | 0 | N | N |
| | H3 | China | Female | 57 | 19.8 | 109.5 | 0 | 0 | 1 | 2 | N | N |
| | H4 | China | Male | 61 | 21.2 | 116 | 0 | 0 | 7 | 11 | N | N |
| | H5 | China | Female | 54 | 20 | 115 | 0 | 0 | 0 | 0 | N | N |
| | H6 | China | Female | 54 | 18.5 | 108.5 | 0 | 0 | 0 | 0 | N | N |
| | H7 | China | Female | 59 | 18 | 108 | 0 | 0 | 0 | 0 | N | N |
| | H8 | China | Female | 63 | 20 | 110 | 0 | 0 | 1 | 1 | N | N |
| | H9 | China | Male | 58 | 21 | 112 | 0 | 0 | 0 | 0 | N | N |
| | H10 | China | Male | 68 | 20.8 | 117 | 0 | 0 | 0 | 0 | N | N |
| Caries free | H11 | China | Male | 66 | 18.8 | 109 | 0 | 0 | 0 | 0 | N | N |
| | H12 | China | Male | 66 | 28 | 105 | 0 | 0 | 0 | 0 | N | N |
| | H13 | China | Female | 68 | 19 | 110 | 0 | 0 | 0 | 0 | N | N |
| | H14 | China | Female | 67 | 20 | 115 | 0 | 0 | 2 | 2 | N | N |
| | H15 | China | Male | 60 | 24.2 | 119.5 | 0 | 0 | 0 | 0 | N | N |
| | H16 | China | Male | 64 | 26 | 120 | 0 | 0 | 0 | 0 | N | N |
| | H17 | China | Female | 67 | 22 | 115 | 0 | 0 | 1 | 1 | N | N |
| | H18 | China | Male | 74 | 19.6 | 109 | 0 | 0 | 0 | 0 | N | N |
| | H19 | China | Male | 67 | 18 | 109.5 | 0 | 0 | 0 | 0 | N | N |
| | H20 | China | Male | 71 | 17.5 | 108 | 0 | 0 | 0 | 0 | N | N |
| | H21 | China | Male | 67 | 18.5 | 110 | 0 | 0 | 0 | 0 | N | N |
| | C1 | China | Female | 73 | 20.7 | 110 | 9 | 14 | 10 | 15 | N | N |
| | C2 | China | Female | 74 | 19.8 | 109.5 | 12 | 12 | 13 | 38 | N | N |
| | C3 | China | Female | 68 | 18.5 | 108 | 10 | 12 | 12 | 14 | N | N |
| | C4 | China | Male | 71 | 22 | 112 | 15 | 18 | 15 | 21 | N | N |
| | C5 | China | Female | 68 | 19.5 | 111.5 | 11 | 15 | 12 | 34 | N | N |
| | C6 | China | Male | 70 | 18 | 109 | 13 | 32 | 13 | 34 | N | N |
| | C7 | China | Male | 59 | 20.7 | 115 | 11 | 22 | 12 | 23 | N | N |
| | C8 | China | Male | 66 | 20 | 117 | 11 | 14 | 11 | 16 | N | N |
| | C9 | China | Female | 74 | 19.8 | 116.5 | 16 | 35 | 16 | 35 | N | N |
| Caries | C10 | China | Female | 58 | 22.6 | 113 | 14 | 19 | 14 | 19 | N | N |
| | C11 | China | Male | 64 | 20 | 115 | 15 | 23 | 15 | 23 | N | N |
| | C12 | China | Male | 59 | 19 | 113 | 12 | 18 | 15 | 21 | N | N |
| | C13 | China | Male | 59 | 20 | 115 | 12 | 12 | 13 | 16 | N | N |
| | C14 | China | Female | 58 | 16 | 108 | 11 | 15 | 12 | 18 | N | N |
| | C15 | China | Female | 59 | 17.5 | 108 | 10 | 15 | 11 | 18 | N | N |
| | C16 | China | Female | 71 | 21 | 113.5 | 14 | 19 | 14 | 20 | N | N |
| | C17 | China | Female | 59 | 18 | 110 | 11 | 14 | 11 | 16 | N | N |
| | C18 | China | Male | 59 | 18.7 | 106 | 12 | 11 | 14 | 16 | N | N |
| | C19 | China | Male | 58 | 16.5 | 120 | 13 | 49 | 13 | 50 | N | N |
| | C20 | China | Male | 68 | 18 | 114 | 14 | 41 | 15 | 42 | N | N |
