# Supplementary material for: Profiling of Oral Microbiota in Early Childhood Caries Using Single-Molecule Real-Time Sequencing
Source: Front Microbiol. 2017 Nov 15;8:2244. doi: 10.3389/fmicb.2017.02244 (PMC5694851; doi:10.3389/fmicb.2017.02244)
Supplement: Supplementary file 8 [file Table3.PDF]

Table S3. Data production of 41 samples in our research.

Group	Sample ID	Total bases	Number of sequences	Average length(bp)	Q20	Q30	GC Content(%)
Caries free	H1	49991070	33832	1477.6	93.7	88.24	52.818
	H2	21846888	15132	1443.8	93.56	87.76	52.9161
	H3	27506932	18772	1465.3	93.47	87.64	52.9205
	H4	14682592	9942	1476.8	92.31	85.42	52.7828
	H5	16579814	11374	1457.7	92.35	85.4	52.7315
	H6	16685854	11294	1477.4	92.35	85.51	53.1897
	H7	18199474	12328	1476.3	91.99	84.81	52.9046
	H8	22794132	15542	1466.6	92.09	85.13	52.7207
	H9	15825994	10742	1473.3	92.59	85.93	52.4804
	H10	12985090	8692	1493.9	92.28	85.16	52.8096
	H11	15570128	10656	1461.2	92.47	85.77	52.689
	H12	36930086	25000	1477.2	93.26	87.63	52.8036
	H13	15866046	10772	1472.9	93.1	86.63	52.6618
	H14	17170474	12084	1420.9	93.98	88.58	52.408
	H15	20162386	13700	1471.7	92.31	85.31	52.572
	H16	35040096	23806	1471.9	93.42	87.85	52.795
	H17	17429362	11754	1482.8	92.92	86.29	52.9625
	H18	31096186	21044	1477.7	93.41	87.87	52.586
	H19	19815982	13854	1430.3	94.04	88.54	52.4516
	H20	44136706	29692	1486.5	93.39	87.82	53.0483
	H21	49260212	33310	1478.8	93.7	88.32	52.8171
Caries	C1	39180618	26412	1483.4	93.62	88.23	52.878
	C2	31566864	21612	1460.6	93.49	87.7	52.9661
	C3	28542846	19314	1477.8	93.43	87.67	53.1516
	C4	22877580	15444	1481.3	93.85	88.17	52.784
	C5	31456962	21356	1473	93.52	87.7	52.5833
	C6	25603130	17480	1464.7	93.07	87.01	52.8387
	C7	15888482	11234	1414.3	93.3	87.42	52.8966
	C8	24559904	17046	1440.8	93.63	87.92	52.7455
	C9	25109474	17076	1470.5	93.68	88.02	52.8587
	C10	18854400	13168	1431.8	93.19	87.2	52.8417
	C11	31675010	21452	1476.6	93.76	88.07	52.5612
	C12	21805090	15276	1427.4	93.34	87.36	53.0704
	C13	18757838	13042	1438.3	93.01	86.96	52.8181
	C14	22196492	15554	1427.1	93.31	87.35	52.8461
	C15	33948024	22814	1488	93.7	88.32	52.8724
	C16	20555020	13968	1471.6	93.17	87.13	53.1928
	C17	17794590	12566	1416.1	93.38	87.53	52.9212
	C18	18839974	13114	1436.6	93.04	87.1	52.9448
	C19	37034276	24900	1487.3	93.69	88.3	52.9626
	C20	23830730	16154	1475.2	94.17	88.8	53.0312
