# Supplementary material for: Profiling of Oral Microbiota in Early Childhood Caries Using Single-Molecule Real-Time Sequencing
Source: Front Microbiol. 2017 Nov 15;8:2244. doi: 10.3389/fmicb.2017.02244 (PMC5694851; doi:10.3389/fmicb.2017.02244)
Supplement: Supplementary file 9 [file Table4.PDF]

Table S4. Richness of oral saliva and α - diversity of 41 samples in our research.

Group	Sample ID	0.97					
		OTU	ace	chao	coverage	shannon	simpson
Caries free	H1	587	587	587	1	3.54	0.0896
	H2	380	380	380	1	3.78	0.058
	H3	467	467	467	1	3.28	0.1759
	H4	367	367	367	1	3.99	0.0553
	H5	396	396	396	1	3.77	0.0657
	H6	356	356	356	1	3.91	0.0659
	H7	362	362	362	1	3.5	0.1245
	H8	405	405	405	1	3.73	0.0633
	H9	397	397	397	1	4.2	0.037
	H10	338	338	338	1	4.02	0.0428
	H11	339	339	339	1	3.7	0.0649
	H12	738	738	738	1	4.42	0.04
	H13	404	404	404	1	4.21	0.0344
	H14	392	392	392	1	3.64	0.0971
	H15	439	439	439	1	3.55	0.097
	H16	615	615	615	1	3.95	0.0508
	H17	384	384	384	1	3.59	0.1285
	H18	571	571	571	1	3.87	0.0732
	H19	473	473	473	1	4.15	0.047
	H20	575	575	575	1	3.77	0.078
	H21	525	525	525	1	3.42	0.0893
Caries	C1	695	695	695	1	4.29	0.0638
	C2	553	553	553	1	3.97	0.0588
	C3	484	484	484	1	3.65	0.1132
	C4	531	531	531	1	4.25	0.0405
	C5	565	565	565	1	3.82	0.0766
	C6	294	294	294	1	2.77	0.1738
	C7	361	361	361	1	3.99	0.0417
	C8	544	544	544	1	4.22	0.0384
	C9	561	561	561	1	4.2	0.0399
	C10	359	359	359	1	3.38	0.1221
	C11	526	526	526	1	3.97	0.0527
	C12	404	404	404	1	3.49	0.1088
	C13	438	438	438	1	3.94	0.0665
	C14	504	504	504	1	3.94	0.071
	C15	667	667	667	1	4.09	0.0495
	C16	395	395	395	1	3.37	0.1778
	C17	436	436	436	1	4.22	0.0584
	C18	396	396	396	1	3.98	0.0543
	C19	538	538	538	1	3.49	0.1001
	C20	428	428	428	1	3.74	0.0601
