# Supplementary material for: Profiling of Oral Microbiota in Early Childhood Caries Using Single-Molecule Real-Time Sequencing
Source: Front Microbiol. 2017 Nov 15;8:2244. doi: 10.3389/fmicb.2017.02244 (PMC5694851; doi:10.3389/fmicb.2017.02244)
Supplement: Supplementary file 10 [file Table5.PDF]

Table S5. Core microbiome in caries and caries free children.

Genus level	Abundance of core microbiome		
Group / Definition	Caries	Caries free	total
100% core microbiome	0.903660863	0.917766982	0.910885948
95% core microbiome	0.944418524	0.953615699	0.949129272
90% core microbiome	0.952559648	0.961962031	0.957375503
85% core microbiome	0.980279039	0.981400661	0.980853529

Species level	Abundance of core microbiome		
Group / Definition	Caries	Caries free	total
100% core microbiome	0.802831479	0.818794504	0.811007663
95% core microbiome	0.845365167	0.859604958	0.852658718
90% core microbiome	0.873209484	0.882351207	0.87789183
85% core microbiome	0.907081168	0.916260286	0.911782667
